# Sustainable Growth and Synchronization in Protocell Models

**DOI:** 10.3390/life9030068

**Published:** 2019-08-21

**Authors:** Roberto Serra, Marco Villani

**Affiliations:** 1 Department of Physics, Informatics and Mathematics, Modena and Reggio Emilia University, Via Campi 213/A, 41125 Modena, Italy; 2 European Centre for Living Technology, Ca’ Bottacin, Dorsoduro 3911, Calle Crosera, 30123 Venice, Italy; 3 Institute for Advanced Study, University of Amsterdam, Oude Turfmarkt 147, 1012 GC Amsterdam, The Netherlands

**Keywords:** protocell, synchronization, replication, reproduction, Gillespie algorithm, autocatalytic set, RAF set

## Abstract

The growth of a population of protocells requires that the two key processes of replication of the protogenetic material and reproduction of the whole protocell take place at the same rate. While in many ODE-based models such synchronization spontaneously develops, this does not happen in the important case of quadratic growth terms. Here we show that spontaneous synchronization can be recovered (i) by requiring that the transmembrane diffusion of precursors takes place at a finite rate, or (ii) by introducing a finite lifetime of the molecular complexes. We then consider reaction networks that grow by the addition of newly synthesized chemicals in a binary polymer model, and analyze their behaviors in growing and dividing protocells, thereby confirming the importance of (i) and (ii) for synchronization. We describe some interesting phenomena (like long-term oscillations of duplication times) and show that the presence of food-generated autocatalytic cycles is not sufficient to guarantee synchronization: in the case of cycles with a complex structure, it is often observed that only some subcycles survive and synchronize, while others die out. This shows the importance of truly dynamic models that can uncover effects that cannot be detected by static graph theoretical analyses.

## 1. Introduction

How precisely the transition from an abiotic environment to one that hosts life took place is a major scientific question that is still in need of a solution. While it may turn out to be actually impossible to retrace in detail all the steps that led, about 3.5 billion years ago, to the appearance of the first living beings on earth, there are general questions that need to be addressed by theoretical and experimental research, the most prominent one concerning how “generic” life is (see e.g., the classical works of Kauffman [1] and Monod [2] for two very different views). Is its emergence very likely, or even unavoidable, in a suitable environment, or is it due to an extremely improbable set of accidents?

In this paper we will suppose, as a working hypothesis, that there is a finite probability that life will appear when certain conditions are met. This hypothesis is also relevant for the study of possible life forms in other parts of the universe, as well as for the feasibility of developing lifelike forms in the laboratory.

Of course, in order to fully address this topic, one needs a definition of “living,” a notoriously difficult and subtle problem. Following Rasmussen et al. [3], in this paper we will limit our attention to thermodynamically nonisolated sets of entities that are

- able to grow, building part of their own elements using building blocks from their environment;
- similar but not necessarily identical to each other;
- able to give rise to descendants, which are on average more similar to their parents than to the rest of the population.

We will also suppose that such entities grow in a controlled environment like a chemical laboratory, where some necessary conditions for their growth and reproduction can be managed from outside. We will therefore avoid any discussion about the place where life on earth might have started. Note that, if the size of the population of entities is limited in some way, then the previous conditions open the way to a kind of evolution, where fitter individuals, or groups of individuals, can displace poorly performing ones. It is a question of wording, with little relevance, whether one calls this “chemical” or “biological” evolution.

It should be stressed that more stringent definitions of life have been proposed (see, e.g., [4,5,6]). We are aware that meeting the above conditions (i)–(iii) might not amount to a final word about the possibility of true abiogenesis, yet we believe that achieving such a goal would be a major breakthrough, which might even help us to better define life.

Given that we take as our working hypothesis that life is to some extent generic, we must analyze it with quite generic concepts and models. Note also that, in this case, mathematical and computational models [7,8,9] are even more important than usual, given that we do not yet have experimentally available protocells fully endowed with the properties described above.

Indeed, several proposals have been suggested for how protocells might be made, concerning both their overall “architecture” and organization and the chemical nature of their parts. We will not review here the extensive literature, referring the interested reader to books or other reviews [9,10,11,12,13]. We will present here quite abstract models that can correspond to several more detailed proposals and we will look for the main dynamic properties of these models. We will suppose that a protocell is made up of at least a “container” and a set of “replicators” (chemical species that can (under certain conditions) collectively self-replicate—a species capable of directly replicating itself will be called a self-replicator), aka genetic memory molecules, or GMMs for short. We will not make explicit statements about the kind of chemicals that make up the container, but in general we have in mind lipid vesicles (composed of an aqueous core surrounded by a lipid bilayer), which are spontaneously formed by amphiphiles in water under a wide set of conditions.

Vesicles are known to undergo fission [11,14,15,16]. For the sake of simplicity, we will assume that a vesicle is spherical, and that it suddenly divides into two approximately equal parts when it reaches a certain size. Therefore, the growth rate also determines the reproduction rate. Note that our models are unable to describe the actual fission process [17,18,19], whose duration is supposed to be much shorter than that of the protocell growth.

We will also not make any explicit comment on the nature of the GMMs: although it is often assumed that they are nucleic acids with template-based replication (e.g., in the RNA world hypothesis [20]), we will assume in the first part of the paper quite general kinetic equations. In the second part of the paper we will make more focused hypotheses, which are closer to the spirit of the “polypeptide first” approach—although even there no specific properties of peptides will be claimed.

Our models are composed of two sets of dynamical evolution rules, one describing the growth of the lipid container and the other describing the reactions among the GMMs. We will assume that these two sets of chemicals are coupled, i.e., that some replicators affect the growth (and therefore the reproduction) rate of the whole protocell (otherwise the two dynamical processes would be independent, and no true protocell would exist).

Note that two growth and duplication processes, i.e., the replication of the GMMs and the reproduction of the container, take place in these protocells. A necessary condition for the sustainable growth of the protocell population is that the two processes take place at the same rate, i.e., that they synchronize. Our emphasis on synchronization does not exclude the possibility of different behaviors, like the occasional fission of a protocell, but is related to the request for sustainable growth of a population of protocells, which is a necessary precondition for evolution. The kind of synchronization that is studied here can take place in a constant external environment, while other interesting behaviors might take place due to time-varying external conditions, e.g., those hypothesized by [21].

As described in detail in [22], synchronization means that, after an initial transient, the pace of fission of the lipid container equals that of the duplication of the genetic material so that the concentrations of replicators become the same for every replication cycle. Here it is assumed that the protocell divides into two equal daughter cells when its size reach a fixed threshold. (Let us mention that we have also tested the robustness of our results by introducing stochasticity in some simulations, where the splitting threshold size is subject to fluctuations, and where the two daughter cells may randomly differ [13].) The importance of synchronization has been stressed by [23] in the specific case of the so-called Los Alamos bug model. In previous works [13,22,24,25,26,27,28,29], we have extensively discussed several models, and we have been able to show that the rates of the two processes tend to a common value, generation after generation, under a wide set of hypotheses concerning either the type of kinetic equation or the protocell architecture. So, synchronization is a widespread, emergent property. However, there are also interesting cases where such synchronization does not take place; in these cases, either the concentration of some GMM can grow unbounded, or the concentrations of all the GMMs approach 0.

The models above, like those of Section 2 below, are based on deterministic differential equations; supposing that both the aqueous interior and the lipid membrane are internally homogeneous, one can make use of ordinary differential equations (ODEs). Looking for quite generic properties, it is necessary to consider various models, with different kinetic equations, and to verify how robust to various changes a given property is.

In Section 2 we will quickly summarize the main outcomes of our previous studies, referring to the original papers for details, and we will consider in particular the so-called “internal reaction models” (IRMs for short), where the relevant chemical reactions (i.e., replication of the GMMs and synthesis of new lipids) take place in the interior of the protocell, which is not directly available to the external environment. The protocell membrane is supposed to be semipermeable, so that some chemical species can cross it.

The external environment is supposed to be an infinite beaker, where some chemical species are kept at constant concentrations. In order to keep the model as simple as possible, semipermeability will be described as an all-or-none (i.e., Boolean) property, and some of the chemicals present in the external environment will be able to cross the membrane. Both the external beaker and the internal protocell volume will be assumed to be homogeneous, i.e., the diffusion of solutes is fast enough to allow us to ignore spatial inhomogeneities in the two aqueous phases.

In the simplest versions of the model, transmembrane diffusion is assumed to be instantaneous. In this case, when the replication equations are quadratic (an important case indeed), there is no spontaneous synchronization (see Section 2 for details). If, however, we adopt a more realistic viewpoint, and if we assume a finite transmembrane diffusion rate (TDR), then synchronization can actually take place for some wide range of parameter values. This observation highlights the importance of seemingly innocent hypotheses that can affect the behavior of complex models like those we consider here. Indeed, pointing out the importance of the finiteness of the TDR in studying synchronization in nonlinear systems is a major result of this study.

It seems that the lack of synchronization of the quadratic case is related to the fact that some processes become too fast—transmembrane diffusion being an example. Another important example is the formation of intermediate complexes with a finite lifetime. Suppose, for example, that the macroscopic kinetic equation for species X is dX/dt = kXY, so the formation of new Xs is proportional to the frequency of encounters between X and Y. However, this is shorthand for a two-step process in which X and Y bind together, forming a “complex” that later gives rise, from suitable precursors, to a new X molecule. The whole process (neglecting precursors) is X + Y → X:Y → X + X + Y.

If we analyze the kinetic equations (with an infinite diffusion rate through the membrane), we find no synchronization, either by analytical means or by simulations. If we simulate the steps of the reaction, including complexes, we observe synchronization for some range of parameters if their lifetime is finite. In the limit where their lifetime tends to 0, we observe a paradoxical result, i.e., the concentration of some chemicals tends to diverge—an outcome that can be considered a kind of nonsynchronization.

It is extremely important to stress that in several cases it is not necessary to finetune all parameters, since synchronization may well be a spontaneous process—provided that we take care to avoid oversimplifications, which, in certain cases, can make the pace too fast.

Note that the conditions for synchronization that can be obtained using the methods of Section 2 assume that the replicators are already there. Some of them may become extinct, and some may be regenerated at some point in time, but in any case they are there, in the sense that we can write ab initio the complete set of kinetic equations. However, it is highly likely that replication and reproduction were not present from the very beginning, but that they appeared only after some new molecular types had been created by chemical reactions among the initial species. Describing this type of processes is so important that the study of OOL has largely been focused, following the seminal works by Oparin [30,31] and Haldane [32], on the search for the conditions that allow the replication of a set of molecules. However, it is well known that all living beings are composed of cells, so we will keep our focus on protocells and their dynamics. Having said that, the rise of molecular self-replication still remains a key part of the problem—but it has to be described in a protocell.

As sketched above, we imagine that our protocell is surrounded by a bulk solution. The approximately spherical membrane of the first protocell(s) spontaneously forms from pre-existing amphiphiles (referred to also as lipids), surrounding a portion of the bulk fluid (here we assume that the membrane simply surrounds an existing portion of bulk fluid; some studies [33,34] suggest that the formation of a vesicle can alter the chemical environment, thus making the interior different from the rest of the fluid. In this case, a new hypothesis should be considered, besides those mentioned here). In the first generation of protocells, it will therefore be assumed that some impermeable species can be found in the internal phases of protocells. Then some reactions take place inside the protocell, creating new replicators and new lipids. However, if the protocell is large and the concentrations are not too low, the chemicals in the interior of the cell are just like those in the bulk of the solution: what happens inside is equal to what happens in its external environment. Replication pops up everywhere, but this does not seem to be a realistic case.

There are two alternatives:

(a) protocells are small
(b) membranes play active roles

(a) If protocells, bounded by semipermeable membranes, are small, then their internal initial compositions may be very different from the bulk due to statistical fluctuations. For example, the average number of molecules of a chemical with a concentration of 1 μM, which are found in a vesicle whose diameter is 100 nm, is only 0.1 [13]: one molecule in 10 vesicles! The fact that some species in the bulk are absent in a cell makes its chemical environment different from the bulk, and different species can be synthesized. Note also that the initial compositions of different vesicles will be different, thus allowing parallel exploration of different possible chemical futures.

(b) If protocells are large, with linear dimensions of say 10 μm, then the concentration fluctuations may be small and essentially irrelevant. In this case, the internal environment can differ from the external one if the vesicle membrane plays an active role, e.g., either by directly catalyzing some reactions, or by creating a local environment near their borders that favors or prohibits some reactions [29]. The same should happen both inside and outside the cell, but in the latter case diffusion quickly removes the reaction products, while on the other side they are bound to remain in the interior. Of course, membranes might do also this in small vesicles.

If collective self-replication of the GMMs appears in some vesicles at some point in time, after the “discovery” of some new molecular species, then it is likely that the newcomers will be present in small copy numbers, while the concentrations of others are much larger.

Tackling that aspect requires some change with respect to the ODE-based approach of the first part of the paper, i.e., it calls for the use of truly stochastic methods. The Gillespie algorithm [35,36] is a well-known and widely used method to deal with stochasticity in homogeneous chemical reaction systems: it is an asynchronous algorithm, which at every time step assigns a probability distribution to the possible reactions, and draws at random the one that actually takes place. The probabilities depend on the kinetic constants, on some parameters like the temperature, and also on the concentrations of the various chemicals. Since the concentrations change over time, the probabilities do also. Moreover, the effective duration of the time interval is also updated at every step [37]. The Gillespie algorithm has proven effective at dealing with stochasticity in chemical reaction systems with different concentrations, and it will be used since the concentrations of the various species can differ by several orders of magnitude. We guess that this is not going to introduce any significant bias in our study. In order to speed up the computation time, we also performed some deterministic experiments with a simplified procedure (introduced by [38]), i.e., using the standard kinetic ODEs when the concentrations are higher than a threshold, and setting them to 0 when they reach the threshold. The results obtained in this way have usually been double checked, on a subset of cases, by comparing them with truly stochastic simulations.

In order to deal with the appearance of new species, it is also necessary to provide a model for their creation (and destruction). Unfortunately, any choice of a molecular model necessarily introduces “idiosyncratic” hypotheses and makes some transformations possible, others difficult, and still others impossible. We see no way to avoid the limitations that are associated with such a choice. In this paper, we will model our chemicals and reactions on the random binary polymer model (BPM) introduced by Kauffman [39], the behavior of which can be explored by simulations. An attractive feature of this model is that it can describe the emergence over time of longer and longer molecules, and life as we know it is indeed based on large polymers. In future works, it will be interesting to consider the robustness of some of our results when models different from the BPM are used.

A relevant feature of the binary polymer model (BPM), which also mimics what happens in chemical reactions in cells, is that it is concerned with catalyzed reactions only (variants have been proposed where a few reactions can spontaneously take place, at a very small but nonvanishing rate, without catalysis). The need for catalysts has important kinetic consequences, as it requires the encounter not only of the reactants, but also of the catalyst. For example, catalyzed cleavage reactions (which cut a polymer into two) are bimolecular, and catalyzed condensation reactions (which glue together two polymers to form a larger one) are three-molecular. Since three-molecular encounters are very rare, condensations are described as a series of two bimolecular reactions: the formation of a metastable intermediate complex, made by one of the two reactants and by the catalyst, followed by the formation of the final product when the complex encounters the second substrate. As we shall see, the hypotheses about the lifetime of enzyme-substrate complexes can play a major role in determining the behavior of the reaction system.

Since only catalyzed reactions take place at an appreciable rate in the BPM, the sets of molecules that are collectively able to self-replicate are called autocatalytic sets (briefly, ACSs).

The polymer model is reviewed in Section 3. Note that its dynamical behavior depends upon the environment in which the reactions take place. In the past, we studied the formation of ACSs in different environments [40,41], which mimic some features of a dividing protocell: in both cases the concentrations of chemicals that are neither incoming from outside nor produced in its interior tend to vanish over time. So both flow reactors and growing and dividing protocells are able to select those chemical species that are internally produced, using “food” molecules coming from outside. However, they are not identical. A particularly significant difference is that the inflow in a flow reactor is determined from outside, while the internal reactions play no role. On the contrary, in a semipermeable protocell the inflow of permeating species depends upon the concentration differences between the internal water phase and the external environment, and therefore depends upon what is happening inside the protocell. Moreover, in a flow reactor all the chemical species exit in the outflow, while in a semipermeable cell some remain trapped inside.

Therefore, the next obvious thing to do is to insert the dynamical model of replicators in a stylized model of the whole protocell (described in Section 4) [13,28,40,42], which in turn is placed inside an infinite homogeneous system. We do this in Section 5 by using a protocell model, similar to the one adopted in Section 2, supposing that short polymers can freely cross the membrane, while the crossing of longer polymers is absolutely prohibited. We shall see that ACSs, where they come into existence, can lead to synchronized growth and replication of a population of protocells, in a way similar to what had been observed using the ODE formalism of Section 2.

We will find some interesting similarities among the behaviors of these two classes of models (ODE vs. BPM), the major one concerning the effect of a finite transmembrane diffusion rate. In the ODE approach, it was proven that it can allow synchronization even when the interactions are quadratic. In the BPM framework, we will see that finite diffusion allows the coexistence of autocatalytic sets with different growth rates, which is impossible in the case of instantaneous diffusion.

However, the approach based on stochastic simulations of BPMs also provides information about behaviors that are not observed in the deterministic ordinary differential equations. Indeed, the interactions of different ACSs can lead to some counterintuitive outcomes: the most interesting that have been discovered so far are discussed in Section 5. It is worth mentioning that the coupling of the dynamics of the ACS to that of the whole protocell can lead to some sort of selection when there are complex ACSs, composed of subsets that are ACSs themselves: in these cases, one often finds that only some subsets can survive, while others get extinguished. Moreover, in some cases there is no synchronization at all. As will be discussed, these facts show the importance of truly dynamic studies that provide more information than static graph theoretical analyses.

Section 6 is devoted to highlighting some key observations and thoughts about the main modeling problems that are still open, and possible future directions of research. Note that in Section 3 and Section 4 we review the known results; they are here in order to make the paper complete, and they do not report new results or hypotheses, which can be found in the other sections.

## 2. Synchronization in ODE Models of Protocells: Results and Discussion

Let us consider a spherical vesicle whose membrane is composed of amphiphiles, with an aqueous interior, which is placed in an infinite beaker. Let us suppose for simplicity that in the membrane there is a single kind of amphiphile whose quantity is C, and that in the internal water phase (whose volume is V) there is a single kind of replicator (or GMM) whose quantity is X. Square brackets are used to indicate concentrations in the internal water phase, where the key reactions are supposed to take place. Let Pc and Ps denote precursors of lipids and GMMs, respectively, which are supposed to be able to cross the membrane. Then, following the law of mass action, the macroscopic kinetic equations are [22,24,26]:

(1) $$\frac{dC}{dt}=\alpha {}^{\prime }Vf\left(\left[X\right],\left[P_{C}\right]\right)+\chi V\left[P_{C}\right]-\varphi \left(C\right)\;\frac{dX}{dt}=\eta {}^{\prime }Vg\left(\left[X\right],\left[P_{S}\right]\right)-\psi \left(X\right),$$

where we have assumed that *X* can affect the lipid growth rate, and where *φ*(*C*) and *ψ*(*X*) denote spontaneous decay terms, while *f* and *g* are model-dependent functions. We suppose that the geometry is fixed, so there is a fixed relationship between the internal volume *V* and the volume of the membrane, which in turn is proportional to *C*. The cell grows until it reaches a threshold size, at which point it breaks into two equal “daughters”: each one inherits one-half of the lipids, and both have the same volume—which can be smaller than one-half of the mother’s volume [28] (see also the Supplementary Materials):

(2) $$V_{daughter}=\frac{\xi }{2}V_{mother},$$

where *ξ* < 1. If both mother and daughters are spherical, then *ξ* ≅ 0.707 (see the Supplementary Materials).

Let us suppose that, in the growth phase, before halving, the decay terms play no significant role, and that the noncatalyzed growth of lipids is also negligible. As discussed in the Introduction, here are two major model alternatives:

(a) one can assume infinitely fast diffusion, so that the permeating species Pc and Ps are kept at constant values (equal to those of the infinite beaker where the protocell is placed); or
(b) one can assume that the precursors cross the membrane at a rate proportional to their concentration differences between the outside and the internal environments (i.e., according to Fick’s law).

Let us first consider the first alternative. In this case, only the first terms on the right-hand side of Equation (1) play a role in the continuous growth before halving. If the kinetic rules are assumed to be linear for both lipids and replicators, then the equations take the simple forms

(3) $$\frac{dC}{dt}=\alpha X\;\frac{dX}{dt}=\eta X.$$

As shown in [22], these can be directly integrated. We suppose that each replication cycle ends at size 2*C*_0_, and that it begins at *C*_0_. This allows us to determine *X_final_* (just before halving) from *X_initial_* (just before the start of the growth). At the *k*-th iteration, *X_k_* = *X_initial_* and *X*_*k*+1_ = *X_final_ξ*/2, which provides an iteration map for *X*_1_*, X*_2_, etc., synchronization corresponds to the *X_s_* growing at the same rate as the protocells, i.e., *X_k_* tends to a constant value as *k* → ∞, and one can directly verify that this happens in the case of Equation (3). So synchronization is a spontaneous, emergent property in these systems; there is no need to finetune the kinetic parameters to achieve synchronization (note that synchronization is an asymptotic property, which is not achieved with an infinite precision in finite time; as usual, we will assume that it has actually been achieved when the differences in concentrations between two successive generations are negligible.)

The linear Equation (3) is somewhat peculiar, so one might wonder what happens if the apparent reaction rates are different, i.e.,

(4) $$\frac{dC}{dt}=\alpha X^{\gamma }V^{1-\gamma }\;\frac{dX}{dt}=\eta X^{\nu }V^{1-\nu }.$$

Equation (3) is a particular case of Equation (4) that is often used in chemical kinetics (it is usually a shorthand that summarizes the overall effect of more detailed reaction steps [27]).

In order to simplify the analysis, it is possible to change the variables, e.g., by posing

$$d\tau =\alpha X^{\gamma }V^{1-\gamma }dt.$$

Thus *dC*/*dτ* = 1 so, between *τ_k_* and *τ*_*k*+1_, C grows linearly with *τ*, and all the intervals [*τ_k_*, *τ*_*k*+1_] have the same size *C*_0_. One then has

(5) $$C\left(\tau \right)=C_{0}+\tau -\tau_{k}\;C_{final}=C_{0}+\tau_{k+1}-\tau_{k}=2C_{0},$$

where the final subscript denotes the end of the growth cycle between *τ_k_* and *τ*_*k*+1_.

The replicator equation, after separating variables, becomes

(6) $$X^{\gamma -\nu }dX=\frac{\eta }{\alpha }V{\left(C\left(\tau \right)\right)}^{\gamma -\nu }d\tau .$$

Let us now integrate Equation (6) between *τ_k_* and *τ*_*k*+1_. Since *V* is a given function of *C*, which grows linearly with *τ*, the integral on the right-hand side can be rewritten as

(7) $$\frac{\eta }{\alpha }\int_{\tau_{k}}^{\tau_{k+1}}V{\left(C\left(s\right)\right)}^{\gamma -\nu }ds=\frac{\eta }{\alpha }\phi ,$$

where *φ* is a constant, which has the same value in every interval [*τ_k_*, *τ*_*k*+1_].

The integral of the left hand side depends upon the value of the parameters. In the particular case *γ* = *ν* − 1 it becomes

$$ln\frac{X\left(\tau_{k+C0}\right)}{X\left(\tau_{k}\right)}=\frac{\eta \phi }{\alpha }\Rightarrow X\left(\tau_{k+C0}\right)=X\left(\tau_{k}\right)e^{\frac{\eta \phi }{\alpha }C_{0}}.$$

Recalling that *X*_*k*+1_ = *X_final_ξ*/2, one gets the iteration map

$$X_{k+1}=\frac{\xi }{2}e^{\frac{\eta \phi }{\alpha }C_{0}}X_{k},$$

which either diverges or vanishes, so there is no synchronization—unless, of course, the coefficient in front of *X_k_* is equal to one, but this is a very peculiar relationship between the parameter values, and it is not an emergent property like it is in the linear case. Let us then suppose that *γ* ≠ *ν* − 1; then the integral of the left-hand side of Equation (6) is

$$\frac{1}{\gamma -\nu +1}{\left[X{\left(\tau \right)}^{\gamma -\nu +1}\right]}_{\tau_{k}}^{\tau_{k+c0}}.$$

Note that the integral on the right-hand side is positive; since *X* is nondecreasing (as can be checked directly from Equation (4)), it turns out that the denominator must also be positive, so

(8) μ=γ−ν+1>0

By equating the integrals of the left- and right-hand sides of Equation (6), and taking into account that *X*_*k*+1_ = *X_final_ξ*/2, one finally gets the iteration map

(9) Xk+1=ξ2(Xkμ+Q)1μQ≡μηϕα.

This map admits a fixed point:

(10) {X∞=(Qb)1μb≡(2ξ)1μ−1.

It can also be proven that this fixed point is stable. Indeed, a fixed point X* of a discrete map *X*_*k*+1_ = *f*(*X_k_*) is stable if |*f*′(X*)| < 1, and this is the case, since here

$$f^{\prime }\left(X_{\infty }\right)={\left(\frac{\xi }{2}\right)}^{\mu }.$$

So, we can conclude that in the case of Equation (4) there is indeed spontaneous synchronization. However, we must recall that this is true provided that condition [8] holds. Suppose now that *γ* = 1, i.e., that the growth rate of the container depends linearly upon *X*. Then the previous condition implies

ν < 2,
(11)

i.e., that the replicator kinetic should be subquadratic. Since some important models (see, e.g., [43] assume quadratic kinetics, this point deserves deeper analysis. Usually, quadratic models involve at least two different types of replicators. Assuming *γ* = 1, and supposing that only one type of replicators is coupled to the lipid growth, the equations are

(12) $$\frac{dC}{dt}=\alpha X\;\frac{dX}{dt}=\eta^{\prime }\frac{XY}{V}\;\frac{dY}{dt}=\eta^{\prime \prime }\frac{XY}{V}.$$

One can directly prove [13] that in this case there is no synchronization, unless a very specific relationship exists between the parameters.

All these conclusions were drawn by assuming that the transmembrane diffusion rate of the permeable precursors is infinitely fast, so the inside concentrations of precursors equal the external ones: this is what has allowed us to obtain simpler models from the more general model described by Equation (1). Let us now relax this hypothesis by assuming that TDR is ruled by Fick’s law, and by assuming that the other equations are of the same type as Equation (12), i.e., quadratic interactions between the replicators and linear interactions with the lipids. We will assume that there are two types of replicators, say A and B, to avoid confusion with the previous equations, and two types of precursors: FA is used to synthesize new A (from encounters with A and B) and FB is used to synthesize new B (from encounters with A and B)

The equations, written using concentrations, are then

(13) $$\{\begin{array}{c} \frac{d\left[A\right]}{dt}=k_{f}\left[A\right]\left[B\right]\left[F_{A}\right]\\ \frac{d\left[F_{A}\right]}{dt}=K_{F_{a}}\left(\left[F_{A}{}^{*}\right]-\left[F_{A}\right]\right)V^{-\frac{1}{3}}-k_{f}\left[A\right]\left[B\right]\left[F_{A}\right]\\ \frac{d\left[B\right]}{dt}=\eta_{f}\left[A\right]\left[B\right]\left[F_{B}\right]\\ \frac{d\left[F_{B}\right]}{dt}=K_{F_{b}}\left(\left[F_{B}{}^{*}\right]-\left[F_{B}\right]\right)V^{-\frac{1}{3}}-\eta_{f}\left[A\right]\left[B\right]\left[F_{B}\right]\\ \frac{d\left[C\right]}{dt}=\alpha \left[A\right] \end{array},$$

where F* are constant outside concentrations, and *V*^−1/3^ comes from the assumption of a spherical shape (see Supplementary Materials, Section S1, for a more complete presentation of the kinetic parameters).

**Figure 1:**
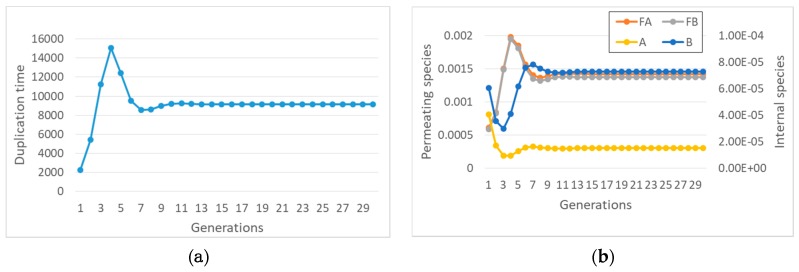
Synchronization in a quadratic model with two replicators and finite transmembrane diffusion rate, described by Equation (13). On the *x*-axis: generation number; on the *y*-axis: (**a**), duplication times; (**b**), concentrations at splitting time-please note that the concentration values are measured at the end of each protocell growth: the initial concentrations of the first generation are all equal to 0.001 M. Parameters are protocell radius r = 1 × 10^−6^ m; membrane thickness δ = 1 × 10^−8^ m; external concentrations F_A_* = 0.01 M and F_B_* = 1 × 10^−2^ M; *k_f_* = 1 × 10^3^ s^−1^M^−2^; *η_f_* = 5 × 10^3^ s^−1^M^−2^; *KF_a_* = *KF_b_* = 8 × 10^−19^, equivalent to a product *D_a_K_a_* = 1 × 10^−12^ m^2^/s, *Da* being the coefficient of diffusion and *K_a_* the partition coefficient. The *K*_F*_ coefficient includes the thickness of the membrane and a form factor due to the shape of the cell. In this work we focus on spherical cells, so it holds that $K_{F_{A}}=\frac{D_{A}K_{A}}{\delta }{\left(36\pi \right)}^{\frac{1}{3}}$(see the Supplementary Materials). The same relationship holds for all the next figures, in which we will only indicate the most indicative product *D_a_K_a_*.

These coupled nonlinear equations can be directly simulated. It is extremely interesting to note that synchronization can indeed be observed (Figure 1), contrary to the case of buffered internal concentrations of precursors.

One might guess that the recovery of emergent synchronization is related to the slowing down of the very fast quadratic kinetics provided by finite diffusion. According to this line of thought, one can also try a different way to slow down the process. Remember that the nonlinear apparent reaction rates actually provide an aggregated description of multi-step reactions. Consider, for example, the equation dX/dt = ηXY/V; it describes a process whereby a fraction of the encounters between X and Y molecules generate new Xs. As we have just seen, this requires the involvement of some other molecules, which are not explicitly described in the equation. So, omitting the extra molecules, the whole process is something like X + Y → X:Y → X + X + Y. Either the formation or the destruction of the complex requires the intervention of other molecules. Moreover, the first step may also be reversible, leading back to X and Y molecules without the creation of any further X. The important point is that the X:Y complex may have a nonvanishing lifetime, and this might slow down the reactions.

The resulting equations, in the case of an infinite transmembrane diffusion rate, are (see the Supplementary Materials Section S1 for a complete presentation of the kinetic parameters):

(14) $$\{\begin{array}{c} \frac{d\left[A\right]}{dt}=-k_{f}\left[A\right]\left[B\right]+2k_{2}\left[A:B\right]\left[\bar{F_{A}}\right]-\eta_{f}\left[A\right]\left[B\right]+k_{2}^{{}^{\prime }}\left[A:B^{\prime }\right]\left[\bar{F_{B}}\right]\\ \frac{d\left[A:B\right]}{dt}=k_{f}\left[A\right]\left[B\right]-k_{2}\left[A:B\right]\left[\bar{F_{A}}\right]\\ \frac{d\left[B\right]}{dt}=-k_{f}\left[A\right]\left[B\right]+k_{2}\left[A:B\right]\left[\bar{F_{A}}\right]-\eta_{f}\left[A\right]\left[B\right]+2k_{2}{}^{\prime }\left[A:B{}^{\prime }\right]\left[\bar{F_{B}}\right]\\ \frac{d\left[A:B^{\prime }\right]}{dt}=\eta_{f}\left[A\right]\left[B\right]-k_{2}^{{}^{\prime }}\left[A:B^{\prime }\right]\left[\bar{F_{B}}\right]\\ \frac{d\left[C\right]}{dt}=\alpha \left[A\right] \end{array}.$$

**Figure 2:**
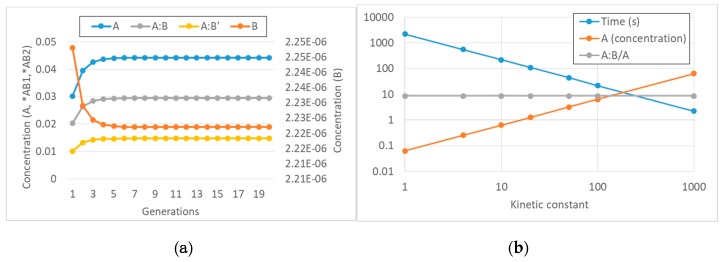
Synchronization in a quadratic two-replicator system with a finite lifetime of the complexes (food species *F_A_* and *F_B_* are internally constant, at 0.001 M). (**a**) Internal concentrations at splitting time (protocell radius r = 1 × 10^−6^ m; membrane thickness δ = 1 × 10^−8^ m; *k_f_* = 2 × 10^3^ s^−1^M^−1^; *η_f_* = 1 × 10^3^ s^−1^M^−1^; *k*_1′_ = 10 s^−1^M^−1^; *k*_2′_ = 10 s^−1^M^−1^). (**b**) Concentration of species A and of the complex A:B at duplication time (at twentieth generation), varying *k*_1′_ = *k*_2′_ from 1 to 1 × 10^3^ s^−1^M^−1^ (log-log scale).

Also, in this case, we checked by simulation what happens: as shown in Figure 2a, there may indeed be synchronization.

The effects of the lifetime of complexes can be understood by considering the effects of an increased rate of disruption (Figure 2b). The feedbacks of the protocell keep the ratio constant between the concentration of a chemical species and the concentration of the complex involved in its formation, whereas the concentrations themselves increase and the duplication time decreases. Therefore, in this case a decrease in the lifetime of the complex leads to unlimited growth of internal concentrations.

The detailed description of the behavior of nonlinear replicators in IRMs, given above (some partial aspects have been published in [13,24,26]), provides interesting information about the behavior of autocatalytic sets in protocells; this will be discussed again in Section 5, where a different model will be analyzed. For the sake of completeness, let us mention a few more results that have already been published in the past (see [13] and further references therein) concerning the synchronization in models with buffered reactants, i.e., infinitely fast transmembrane diffusion.

If there are several replicators interacting in a linear way, the behavior of the model can be related to the behavior of the linear ODE system describing the replicators’ interactions: it is ruled at long times by the eigenvalue of the interaction matrix with the largest real part λ_1_; if Re λ_1_ > 0 the system synchronizes, and the species that survive correspond to the positive components of its eigenvector. In some cases, one asymptotically observes oscillations in time, a phenomenon that has been called supersynchronization (and is compatible with sustained growth of a population of protocells, albeit with nonconstant duplication times—see a similar phenomenon in a nonlinear model in Section 5).

If several replicators interact nonlinearly, then there are different cases. If the nonlinearities are positive quadratic (or higher order) terms, and any of them, say *X_i_X_j_*, appears in the equation for d*X_i_*/d*t* or d*X*_j_/d*t*, then there is no synchronization, thus generalizing the 2D case discussed above. Note, however, that the presence of quadratic terms is not absolutely incompatible with synchronization, a surprising consequence being that even chaotic equations, like the Lorenz system, can indeed synchronize in protocells. The interested reader is referred to [27] for further details about simulations of nonlinear interactions.

Another interesting remark is that similar behaviors are found in protocell models with different architectures, e.g., in surface reaction models like the Los Alamos bug (see [13]).

## 3. Binary Polymer Model

As stated in Section 1, this section summarizes for completeness the random BPM model, without presenting original results. It was introduced by Stuart Kauffman [39] in order to explore the generic properties of systems of chemical reactions involving polymers, which are of course extremely important in biology, and which also are unlikely to spontaneously appear. In present-day cells they are synthesized by starting with monomers, using processes that can increase their length.

So in the BPM the molecular species are composed of monomers that, for the sake of simplicity, are assumed to be of two kinds, say A and B. Obvious generalizations are possible, but here we present the simplest version of the model. Polymers are linear strings of monomers, which are formed by two kinds of reactions: (i) condensation, which glues together two polymers (monomers can be regarded as polymers of unit length) and (ii) cleavage, which cuts a polymer into two shorter ones. It is also supposed that all the reactions that take place are catalyzed, and it is supposed that every polymer has a certain (fixed) probability of catalyzing a reaction. In most cases these probabilities are set to 0, but the probability of catalyzing a reaction is independent of the probability of catalyzing any other reaction: this is certainly a strong hypothesis, very far from actual chemistry, but it has been observed that it is simpler than other hypotheses and it has been argued that it does not compromise the validity of the model [44].

Forgetting for a while protocells, when simulating this model one often assumes that the molecules are placed in either an (infinite) beaker or in a continuous flow reactor. Cleavages are bimolecular reactions, but condensations require the presence of three molecules. Since three-molecular encounters are rare, we chose to model them as a sequence of two bi-molecular reactions as follows [13,41,42]:

1. Cleavage: MN + C → M + N + C   (kinetic coefficient: *k_cl_*)
2. Condensation: (whole reaction: M + N + C → MN + C)
  - Complex formation: M + C → M:C   (kinetic coefficient: *k_cond1_*)
  - Complex dissociation: M:C → M + C   (kinetic coefficient: *k^−1^_cond1_*)
  - Final condensation: M:C + N → MN + C   (kinetic coefficient: *k_cond2_*),

where MN is a polymer composed of two parts, M and N; C is the catalyst; and M:C is a transient complex, which can either get back to M + C or interact with N to generate MN.

In both cases, beaker or flow reactor, some species (the “food”) are assumed to be continuously available (e.g., supplied from the external world). Some polymers may also be present from the beginning, but they are usually quite small ones, the spontaneous generation from outside of long polymers being regarded as unlikely. The attention is then shifted to the appearance of longer polymers as the reactions proceed.

As has been observed in Section 1, the new chemical species may be present at a much lower concentration than some of the old ones; this requires a stochastic treatment that is provided by the Gillespie algorithm, which is asynchronous (a single reaction takes place at each time step, the elementary time step being very small). If we want to represent those species that are there in our system, we cannot make use of only those involved in the chosen reaction. Nor can we, in general, include all the species, since some reactions are so rare that the products they generate are not present at significant levels. The empirical way out of this conundrum is choosing a time window, and considering only the species that are involved in some reactions in the window (unless, of course, they are nonreacting species). The length of the time window sets the length of the system memory. We can provide in such a way a network representation of our system, including only the active species and their reactions, which provides useful information (while depending, of course, upon the length of the window).

It has been observed [39] that, as new polymer types are synthesized, the number of possible reactions increases faster than that of the species. This leads one to think that, as the number of different chemical species is increased, a giant connected component forms in the random reaction network, a consequence being that a set of collectively self-replicating species should appear spontaneously [39,40]. Unless there are catalysts in the food set, this set is bound to be a cycle, i.e., a collectively autocatalytic cycle (ACS), which should spontaneously appear as the diversity of the chemical soup is increased. The importance of cycles in the behavior of chemical reaction networks has been emphasized by several other authors [45,46,47,48].

However, the above reasoning neglects the importance of the dynamics. This was noticed decades ago by Bagley et al. [38,49], who provided the first dynamical simulations of the BPM. Indeed, some reactions may be too rare to have any significant effect. Moreover, a newly formed species can be used by another one as a substrate, and can therefore remain at irrelevant low levels. Dynamical simulations highlight the possible importance of these phenomena, which escape the purview of a static graph theoretical analysis. An important take-home message is therefore that a static study of the properties of the reaction network, based upon analyses of its links (e.g., average degree, cluster index, diameter, betweenness and other topological measures), without taking into account the dynamical processes that take place in the nodes, can be misleading.

Note that the Kauffman model largely relies upon randomness; in particular, every polymer in the system has a probability (which may vanish) to catalyze any possible reaction. Therefore, in different simulations the same species can catalyze different reactions:, we can simulate different “chemistries,” so to speak. This is exactly the language we choose: a set of couples {species, reaction}, where the species catalyzes the reaction, will be called a *chemistry*, since it describes a possible artificial world. We can then simulate different chemistries and look for generic properties of the set of chemistries [40,41,49]; however, in a different series of experiments, we can also keep the chemistry fixed, and simulate various time histories. In principle, these may differ: consider, for example, two small protocells that host inside, at time 0, different sets of replicators, as discussed in Section 5. Note that path dependency might show up even if the two initial sets of replicators are identical, since the discovery of a given catalyst at an early phase in a finite system might channel the following evolution in one way or another.

## 4. Semipermeable Protocell Model

**Figure 3:**
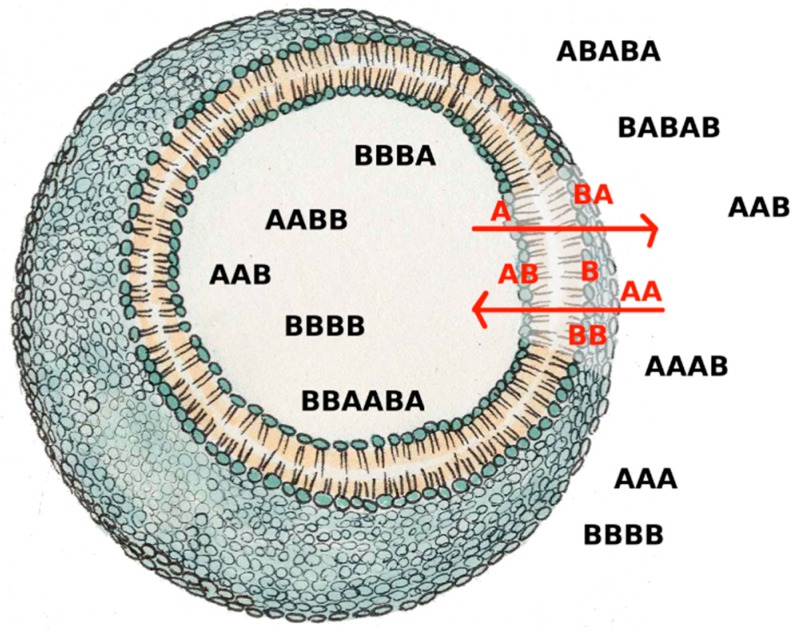
Schematic representation of the semi-permeable membrane as conceived in our model of protocell. The membrane is here represented as a lipid bilayer that shapes a spherical vesicle. In this example, only the species shorter than three letters can cross the membrane.

As mentioned in Section 1, this section summarizes for completeness the main features of the model, without presenting original results. For a detailed presentation, the reader is referred to [13,28,42]. The key reactions are assumed to take place in the internal water phase of the protocell. The semi-permeable membrane is modeled by allowing only some species to enter and leave the protocell, namely those that are shorter than an arbitrary length *L_perm_*. All the species that are longer than *L_perm_* either remain entrapped within the protocell or never enter it from the outside (see Figure 3). Another important assumption is that the concentration of the permeable molecules is homogeneous both inside and outside the protocell, i.e., we assume infinitely fast diffusion in both aqueous phases. As was done in Section 2, we will consider two alternatives:

1. transmembrane diffusion is infinitely fast, so the internal concentrations of the permeable species are identical to the (constant) external ones (i.e., they are buffered);
2. transmembrane diffusion takes place at a finite rate according to Fick’s law, i.e., in a way proportional to the concentration difference of the permeable species and to the surface of the protocell.

As in Section 1 and Section 2, we assume that the protocell lipids can grow and that some internal chemical species *X* can affect the growth of the container, acting as catalysts for the production of membrane lipids. As for the case of the choice of cleavage and condensation catalysts, the species to be associated with the container growth are chosen randomly (often a single species is chosen).

The kinetics of lipid formation are first-order with respect to the concentration of the catalyst, and any lipid produced inside the protocell is assumed to instantaneously become part of the membrane.

Protocells can grow and divide and, as in the previous sections, we will simply assume that protocells are spherical and turgid with constant membrane thickness.

As discussed in Section 1, we take here a simplifying approach to the process of cell division by supposing that there is a fixed threshold value of the size of the protocell, so we assume that when *C* = 2*C*_0_, the protocell divides into two identical daughters. In the division process the molecules present within the protocell at the division moment will be shared in identical proportion between the two daughter protocells. A fraction *ξ* will be lost (i.e., released in the bulk) at division, since we suppose that the concentration is homogeneous in the aqueous phase.

An important point to note is that, due to the growth and fissioning behavior of the protocell, an inert species (i.e., one that does not partake in any reaction) will be diluted generation after generation, until it eventually vanishes. When the concentration of a given polymer gets extremely low, in a continuous approach like that of Equation (1), it approaches 0 and, as has already been remarked, if it falls below a certain threshold (corresponding, e.g., to one molecule in 1 μ^3^) it can be assumed to vanish and be set to 0. In a discrete stochastic model, at the time of division at least a single molecule survives. However, if it is not produced, its effects will essentially be negligible. Moreover, part of the fluid, with its solutes, is released at each division to the outside environment, so in the end the single (or very few) surviving molecule(s) will disappear from the protocell.

## 5. Synchronization in Protocells with Binary Polymers: Results and Discussion

Let us now consider what may happen when new chemical species are synthesized in semipermeable protocells that are growing and dividing, according to the models described above. As before, we will assume that the protocells are placed in an infinite beaker, which contains an aqueous solution with constant concentrations of some chemical species. For the sake of simplicity, these species will be assumed to be polymers (a monomer being a particular case of a polymer) that belong to one of two disjoint sets:

(a) the food set, composed of all the species whose length does not exceed a given value *L_perm_*, which can cross the membrane and do not catalyze any reaction;
(b) the set of replicators, composed of all the species longer than *L_perm_*, which may catalyze some chemical reactions (note that there may be some polymers that do not catalyze any reaction) and cannot cross the cell membrane.

From Section 2, we know that a necessary (albeit insufficient) condition to achieve synchronization is that one (or more) species, coupled to the growth of the container, be continuously synthesized.

This might happen in three different ways:

1. some food molecules catalyze the formation of lipids;
2. there is a chain reaction from the food molecules to the synthesis of a catalyst for lipid formation: food → polymer 1 → ... → catalyst polymer;
3. there is (at least) one cycle of reactions that continuously synthesizes the catalyst for lipid formation using food and other molecules in the cycle.

Case 1 has been ruled out since we assume that food species cannot catalyze any reaction; note also that it could be dealt with using the ODE approach of Section 2. Case 2 is fragile: since food molecules are not supposed to catalyze any reaction, some catalyst must be present ab initio in the protocell in order for the synthesis to proceed. However, if there is no cycle, the first catalysts of the chain are not continuously rebuilt, and they are likely to be diluted in successive generations and eventually die out, leading the chain to collapse. Therefore, we will focus our attention on the third case. A set of reactions and chemical species whose chemical species can be formed by the chemical reactions they catalyze (using themselves as substrates, plus the food species) is sometimes referred to as a reflexive autocatalytic food-generated set (RAF set, or simply RAF for short) in order to stress the importance of the food (for a formal definition, see [50,51]). So a RAF set is a set of reactions and chemical species such that each nonfood species is synthesized by at least one reaction of the set. More precisely, a chemical reaction system is a quadruple {X, F, R, C} where X is a set of chemical species, F ⊂ X is the set of species that are continuously supplied, R is a set of reactions (R:X → X) that take place only if they are catalyzed, and C is a set of catalyst assignments C:X→R. Such a chemical reaction system is a RAF set if every species in X can be generated by a reaction in R, given that all the species in F are present. For a more detailed formal presentation, see [50,51].

**Figure 4:**
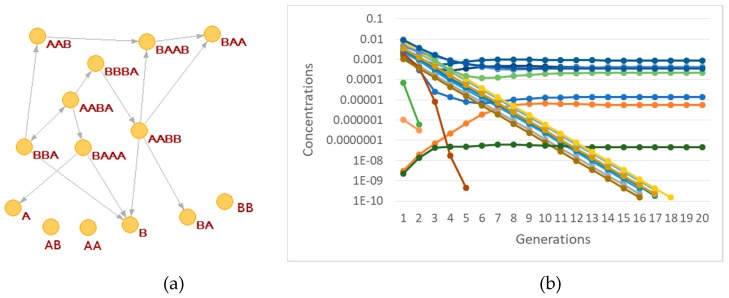
(**a**) A RAF set present within a randomly generated BPM, with the maximum allowed length of the polymers being set to 4 and *L_perm_* = 2. The species not belonging to the RAF are not shown. (**b**) Concentrations at duplication time of the species of the BPM: all the synchronizing molecules belong to the RAF (for clarity of interpretation, the concentrations of the food species are not shown). We remember that (in this plot, and in all the following ones) the concentration values are measured at the end of each protocell growth; in this case the initial concentrations of the first generation are all equal to each other, their value being 1 × 10^−2^ M. (protocell radius r = 1 × 10^−6^ m; membrane thickness δ = 1 × 10^−8^ m; α = 1.0 s^−1^; *D_a_K_a_* = 1 × 10^−12^ m^2^/s; *k_cl_* = 1 × 10^3^ s^−1^M^−1^; *k_cond1_* = 2 × 10^3^ s^−1^M^−1^; *k*^−1^*_cond1_* = 1.486 × 10^1^ s^−1^; *k_cond2_* = 1 × 10^3^ s^−1^M^−1^).

We have already remarked that by using the BPM model it is possible to generate different random chemistries, whereby different polymers catalyze different cleavages or condensations. Depending upon chance and parameter values, in some random chemistries one finds RAF sets. An example is shown in Figure 4a, where the commonly used catalyst-product representation is used (note that this representation, although simple and clear, does not take into account the substrate-product relationships) [13]. Other randomly generated RAF sets show similar behaviors.

In order to understand the behavior of RAF sets, it will be useful to consider two cases:

1. the RAF sets are generated by a random BPM. Note that, if one assumes that protocells are small, it is possible to start the simulations from different sets of initial replicators;
2. the RAF sets are designed in order to highlight some features of their behavior.

If a reaction system gives rise to a RAF set, one of whose components can influence the growth of the membrane, then the protocell can grow and synchronize (an example is shown in Figure 4). Note that all the synchronizing molecules belong to the RAF, while the others are washed away.

The usual definition of a RAF set includes all the species and the reactions that satisfy the RAF conditions. However, it is possible that in the same protocell there are two such disjoint sets or, more generally, that one or more subsets of a RAF are also RAFs; we will thus use the term subRAF to designate a RAF set that is part of a larger one, but retains the RAF property even if the other species are dropped. The importance of the structure of RAF and of the interactions between their parts has been remarked upon by [44,52] in a flow reactor [40] and in protocells [13,42].

In Figure 4 the RAF structure is very simple: the smallest possible subRAF is composed of the species BBA and AABA (plus the foods BB, A. AA and BA). All other species form linear chains of catalyst-product pairs that depend on the autocatalytic action of the pair BBA and AABA.

**Figure 5:**
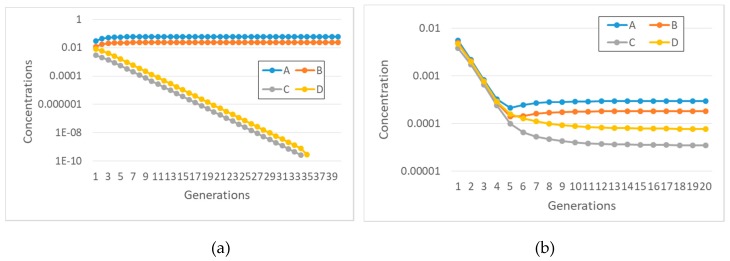
Concentrations at division time of replicators belonging to two independent different subRAFs (A catalyzes the formation of B and B catalyzes the formation of A-the same for C and D) with different growth rates (protocell radius r = 1 × 10^−6^ m; membrane thickness δ = 1 × 10^−8^ m; species A and D are coupled with the membrane, with α = 0.1 s^−1^; only condensations are present, producing the species A, B, C, D, with *k_cond1A_* = 3 × 10^3^ s^−1^M^−1^; *k*^−1^*_cond1A_* = 1.486 × 10^1^ s^−1^; *k_cond2A_* = 1 × 10^3^ s^−1^M^−1^; *k_cond1B_* = 1 × 10^3^ s^−1^M^−1^; *k*^−1^*_cond1B_* = 1.486 × 10^1^ s^−1^; *k_cond2B_* = 7 × 10^2^ s^−1^M^−1^; *k_cond1C_* = 1 × 10^3^ s^−1^M^−1^; *k^−1^_cond1C_* = 1.486 × 10^1^ s^−1^; *k_cond2C_* = 3 × 10^2^ s^−1^M^−1^; *k_cond1D_* = 3 × 10^3^ s^−1^M^−1^; *k*^−1^*_cond1D_* = 1.486 × 10^1^ s^−1^; *k_cond2D_* = 5 × 10^2^ s^−1^M^−1^). All chemical species, including those of the external environment, have the same initial concentration, equal to 1 × 10^−2^ M. (**a**) The case of buffered precursors. (**b**) The same as (**a**), with finite transmembrane diffusion rates (food external concentrations at F*_A_ = F*_B_ = F*_C_ = F*_D_ = 1 × 10^−2^ M, *D_Fa_K_aFa_* = *D_Fb_K_aFb_* = *D_Fc_K_aFc_* = *D_Fd_K_aFd_* = 1 × 10^−10^ m^2^/s).

Let us then consider a case in which two different disjoint subRAFs coexist in the same protocell, in the case of buffered precursors. If the fastest one (i.e., the one capable of increasing the concentrations of its constituents faster, because of its kinetic parameters and/or the characteristics of the food supply) is coupled to the growth of the container, it then synchronizes, while the other one vanishes (see Figure 5a). If the slowest subRAF is coupled to the container, then the fastest RAF grows unbounded, generation after generation; in this case, no synchronization can take place. If both affect the container growth rate, then the fastest RAF survives while the other dies out.

While studying the synchronization of reaction systems described by ODE in Section 2, we found that there is no synchronization when the interactions are quadratic and the transmembrane diffusion is infinitely fast. Synchronization was recovered in that case by limiting the diffusion rate using Fick’s law. Interestingly, the finiteness of TDR also affects the co-existence of different subRAFs (see Figure 5b). This result confirms one of the major observations of Section 2, i.e., that the choice of the diffusion model can greatly affect the conclusions about synchronization.

**Figure 6:**
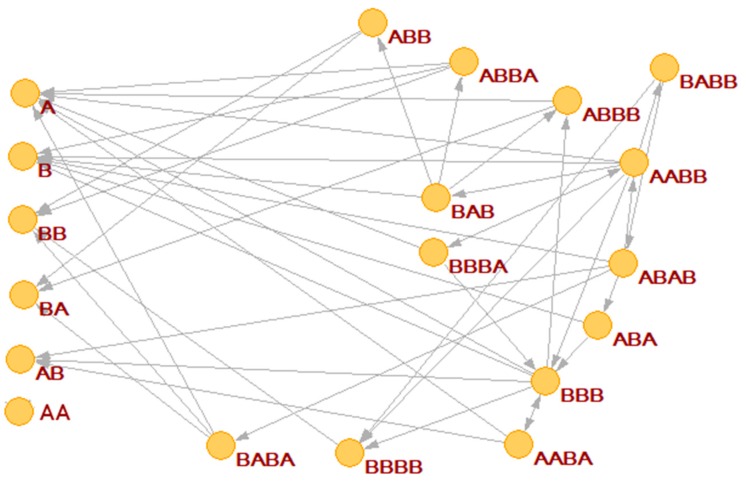
A RAF set present within a randomly generated BPM, with the maximum allowed length of the polymers being set to 4 and *L_perm_* = 2. Molecular species not belonging to the RAF are not shown.

In Figure 6 we present a slightly complex RAF, identified within a randomly generated BPM by means of a graph theoretical study, based on the use of the Steel and Hordijk algorithm [50,53] (therefore we can call it a “static” RAF). When such a RAF is placed in a protocell, and a coupling to the lipid container growth rate is assigned, one cannot take it for granted that all the species in the RAF set will survive. Sometimes only a subset of the whole RAF synchronizes and survives, while sometimes even the dilution of all the chemicals is observed. This result is worth stressing: the interactions with the protocell growth and fission dynamics may actually select some subRAF of the largest theoretically possible RAF. One might speculate about the consequences of this phenomenon in an OOL scenario.

These observations stress the importance of a dynamic analysis whose results may lead to conclusions that are widely different from those suggested by a naive look at the static topology.

**Figure 7:**
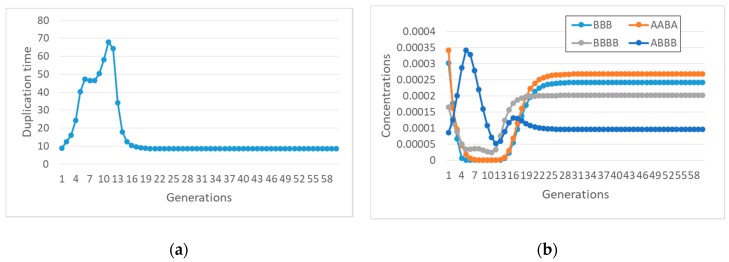
(**a**) duplication times of the system of Figure 4 when species BBBB is coupled to the growth of the container with coefficient α (α = 8 s^−1^ in this simulation). (**b**) concentrations of different species of the overall RAF set of Figure 6-only the four species belonging to the surviving subRAF are shown (protocell radius r = 1 × 10^−6^ m; membrane thickness δ = 1 × 10^−8^ m; *D_a_K_a_* = 1 × 10^−10^ m^2^/s; *k_cl_* = 1 × 10^3^ s^−1^M^−1^; *k_cond1_* = 1000 s^−1^M^−1^; *k^−1^_cond1_* = 1.486 × 10^1^ s^−1^; *k_cond2_* = 1 × 10^3^ s^−1^M^−1^; the external chemical species are buffered at 0.1 M, whereas all internal chemical species have the same initial concentration, equal to 0.001 M).

Let us now consider some specific cases. For example, if species BBBB is coupled to the container, with coupling coefficient α, then one observes synchronization (Figure 7a), but only a small subset of the species in the overall RAF survive (Figure 7b).

**Figure 8:**
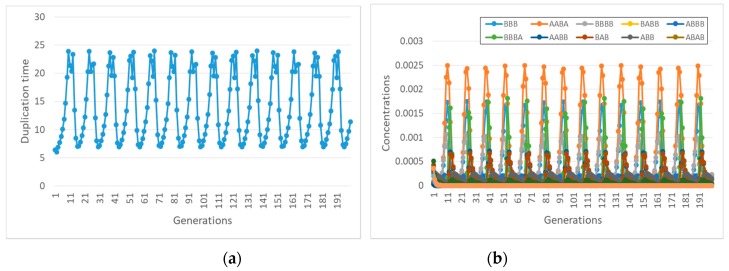
An intriguing type of synchronization, with oscillatory behavior. The reaction system is shown in Figure 6, ABB is coupled to the container growth (α = 8), simulation with finite diffusion rate. (**a**) duplication time; (**b**) concentrations of different species. In this situation, only three species—out of 13 belonging to the complete RAF of Figure 6—disappear (with the same parameters as Figure 7).

It is also important to observe that with different lipids it may happen that different subRAFs survive. Let us consider, for example, what happens to the same reaction system when a different species (ABB) affects the container growth with the same coupling strength α. If the transmembrane diffusion rate of the precursors is finite, then one observed an interesting oscillatory behavior, still a kind of self-sustained synchronization (Figure 8).

**Figure 9:**
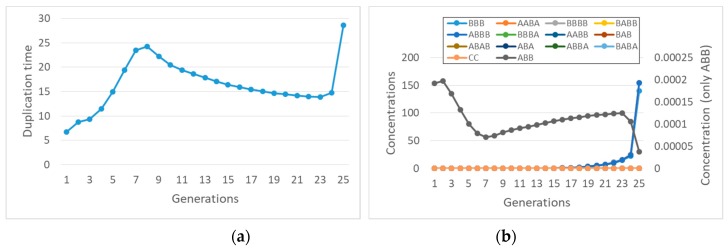
Same system as in Figure 8, but with infinite diffusion rate (and the same coupling coefficient α = 8). (**a**) duplication times; (**b**) quantities of different species. Note that duplication halts while the concentration of the species ABB that can catalyze the formation of new lipids drops. The immediate cause of this decline is the disappearance of the catalyst of ABB production, but the overall process is wider and resides in the feedbacks present in the system (with the same parameters as in Figure 7).

Coming back to the influence of the diffusion rate, let us now suppose, as before, that ABB affects the container growth, but now let the precursors be buffered. In this case, with the same coupling coefficient, starting from an initial condition where all the species are present at the same concentrations, no synchronization is observed (Figure 9a). The duplication time becomes infinite (duplication is stopped) as the concentration of the ABB species drops. This behavior confirms the important role of the transmembrane diffusion coefficient in determining the synchronization behavior of RAFs in protocells.

**Figure 10:**
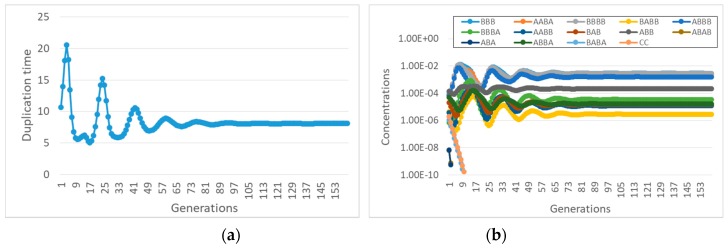
The behavior of the same system as the one shown in Figure 8, but with infinitely fast diffusion rate. (**a**) duplication times; (**b**) concentrations of different species. Remember that, starting from identical initial conditions for all the species, the system dies out, as shown in Figure 9 (with the same parameters as in Figure 7).

Note also that, if we start from a set of initial conditions that correspond to the oscillations of Figure 8, where transmembrane diffusion is finite, and we ideally make it infinitely fast, we observe yet another behavior, this time achieving synchronization at a constant replication rate (Figure 10a).

Another interesting observation is the following. One can generate random chemistries using the BPM (as was done in the case of Figure 4 and Figure 6), using different values for the probability *p* that a randomly chosen polymer catalyzes a randomly chosen reaction. In this case, the average number of links per node of the reaction graph, i.e., its degree, depends upon *p*. Using different values of this parameter, it is then possible to obtain networks with different degrees. Extracting the RAF set (if any) from these networks, one obtains RAFs of different sizes. It is therefore interesting to observe whether they synchronize.

A number of random RAFs of different sizes have been tested, using only those random chemistries that admit a RAF [13], belonging to two sets: one with an average degree <c> = 1, another with degree <c> = 2.5-able to generate larger RAFs. Indeed, the average number of species in each RAF is 2.3 in the first case and 44 in the second. These RAFs have been tested in protocells using three different coupling constants (α1 > α2 > α3), since it has been observed that the fraction of synchronizing RAFs depends upon this parameter. In some cases synchronization was achieved, while in other cases extinction was found. The results are summarized in Table 1.

It is apparent that synchronization is frequently observed in small RAFs, which involve only 2–3 species, while it is much rarer in the case of large RAFs that involve tens of species. The results discussed above give some possible explanations for this seemingly strange result, since competition between subRAFs is more likely to take place in large RAFs than in smaller ones. Note that the improbability of large RAFs’ formation in finite populations has also recently been noted in a different model of prebiotic chemistry, related to the RNA world scenario [54].

**Figure 11:**
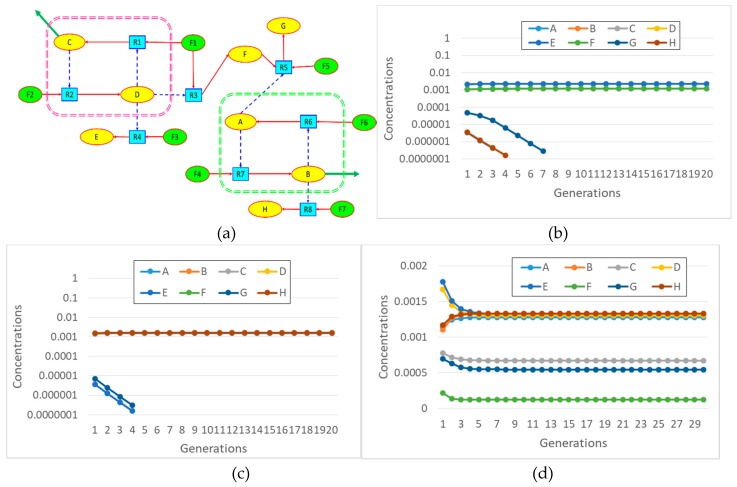
Different asymptotic states reached in semipermeable protocells starting from different initial conditions (protocell radius r = 1 × 10^−7^ m; membrane thickness δ = 1 × 10^−8^ m; *D_a_K_a_* = 1 × 10^−10^ m^2^/s; *k_cl_* = 1 × 10^3^ s^−1^M^−1^; *k_cond1_* = 2 × 10^3^ s^−1^M^−1^; *k*^−1^*_cond1_* = 1.486 × 10^1^ s^−1^; *k_cond2_* = 1 × 10^3^ s^−1^M^−1^; the external chemical species are buffered at 0.001M, whereas all internal chemical species—Including food-have the same initial concentration, equal to 1 × 10^−5^ M) (**a**) The chemistry: the “food” chemical species are represented by means of green ellipses; the internally produced substances by yellow ellipses; the reactions by blue blocks. The presence of two autocatalytic cores is highlighted: the survival of the other chemicals depends (in various modes) on them. It should be noted that the formation of chemical G depends on the simultaneous presence of the two nuclei. (**b**) The synchronizing system in case of initial conditions without species B. (**c**) The same, starting without species D. (**d**) The same, starting without species B and C.

Let us finally come back to a comment raised in the introduction, where it was observed that small semipermeable protocells can start from different initial states and undergo different evolutions. While this is a reasonable hypothesis, it makes sense to verify that this is indeed the case, i.e., that the same chemistry can give rise to different RAF sets starting from different initial conditions. This is shown in Figure 11, where a specific chemistry is drawn and three different asymptotic states are shown that are reached by starting from different initial conditions.

## 6. Conclusions

In this section we will summarize some specific new results, described in Section 2 and Section 5, which seem to us particularly interesting. We will also review a few general lessons learnt and sketch some directions for future research. We will not insist on the importance of synchronization, which has been stressed elsewhere [13,22,24].

### 6.1. Specific Results

A major remark concerns the importance of considering finite transmembrane diffusion rates, either in the ODE models of Section 2 (where they can induce synchronization even in cases where it would not take place in buffered models), or in those of Section 5, where they can allow the co-existence of subRAFs, which would otherwise be incompatible. As we have seen, similar remarks apply when taking into account the finite lifetime of some reaction intermediate complexes.

We regard the case of quadratic interactions as particularly important, since several important and widely used models are of this kind (see, e.g., [43]).

Other noteworthy results concern the possibility of oscillatory phenomena in models of nonlinear replicators, and the importance of the replicator species that are coupled to the growth of the lipid container, since different choices may lead to very different asymptotic states.

### 6.2. General Results

The present study clearly shows the importance of using different kinds of models (like those of Section 2 and Section 5) in order to understand different features of a complex phenomenon like the growth and fission dynamics of protocells.

A point that is worth stressing, in these days when great emphasis is placed on the topological properties of complex networks, concerns the importance of dynamical interactions, which can select some subRAFs among those that can be identified with graph theoretical methods. It is important to note that static analyses alone can be severely misleading, and therefore it is necessary to take dynamics into account.

It is also important to observe that it is possible to achieve different asymptotic states by starting from different initial conditions, which shows that small semipermeable protocells can run the simulation of several subsets of a single chemistry in parallel, thus opening the way to massive experimentation. Last but not least, path dependency can also be observed in these systems.

### 6.3. Indications for Future Work

Several promising future directions of research can be envisaged. We will only mention a few major ones here.

It would definitely be interesting to compare our results to those of models of generation/destruction of species that differ from the BPM, in order to verify how general the present indications are.

In our studies we have considered a simple protocell with a fixed geometry (except for a short time when fission occurs, which is not explicitly described). There are other interesting models where the lipid container grows in a way similar to the one discussed here, but where the volume may have a different dynamics (governed, e.g., by osmosis), so that there is no fixed relationship between membrane size and protocell volume [55].

The models considered here provide conclusions that can in principle be applied to populations of protocells [56], but they do not take into account the true populations, with possible interactions between different cells—this would be a very interesting development.

No less interesting would be to consider what may happen to populations of protocells placed in an “interesting” external environment, e.g., in porous rocks with flows and concentration gradients, instead of a static beaker.

## Figures and Tables

**Table 1:** Fraction of synchronizing RAFs (over a total of 20 observed: α_1_ = 10α_2_ = 100α_3_).

	Fraction of Synchronizing Cases (over 20 Chemistries)		
	α_1_	α_2_	α_3_
<c> = 1, <nRAF> = 2.1	12/20	19/20	19/20
<c> = 2.5, <nRAF> = 43.8	1/20	2/20	2/20

## Supplementary Materials

Some materials are available online at https://www.mdpi.com/2075-1729/9/3/68/s1, The supplementary material section S1 describes the ideas behind the simulations described in Section 5 of the paper; section S2 describes the stochastic simulator; section S3 describes the stochastic simulator and contains the MIT software license. The software source code and some sample of the initialization files are present at the link: http://morespace.unimore.it/marcovillani/software/.
