# Supplementary material for: Sustainable Growth and Synchronization in Protocell Models
Source: Life (Basel). 2019 Aug 21;9(3):68. doi: 10.3390/life9030068 (PMC6789472; doi:10.3390/life9030068)
Supplement: Supplementary file 1 [file life-09-00068-s001.zip › 8.Supplementary/7.life-565834-supple-proofed.zip!/SustainableGrowth_supp_mat_MV2.pdf]

Supplementary material S1

The supplementary material sections (S1, S2 and S3) describes the ideas and the software used to perform the simulations described in Section 5 of the paper. Section C includes the software license.

A general introduction to the software

In this part of the supplementary materials we present some details on the software used and on the simulations. We need to model two main interacting subsystems: (i) the chemical reactions dynamics happening inside the container and (ii) the container itself.

The exchange with the external environment is ruled by the container properties, which could be either a CSTR (a continuous stirred-tank reactor, not analyzed in this paper) or a protocell membrane. For efficiency purposes the container growth are modeled by means of ODEs, whereas within the internal physical space the dynamics of the chemical reactions (ruled by the mass action law) is modeled by means of two different frameworks.

The **first framework** consists on the integration of the differential equations describing the system. The equations are integrated using an Euler algorithm with variable step, already used in [43]. Each chemical can be substrate or product of none, one or more reactions (depending on the chosen artificial chemistry), its overall concentration change being the sum of all concentration changes caused in each reaction in which the chemical is involved. In this case the integration with the container (CSTR or permeable membrane) is straightforward.

In order to present the nomenclature used in the main text, we can concisely expand the cases discussed in Eqs. 13 and 14 of the main text.

Eq. 13:

Table S1 Description of the physic/chemical situation supposed in eq.13 (see the main text)

Physic/chemical situations	Description	Parameters
**Internal reactions**	$A+B+F_A \rightarrow 2A+B$	k_f
	$A+B+F_B \rightarrow A+2B$	η_f
**Interactions with the container**	F_A can pass through the membrane	K_{Fa}
	F_B can pass through the membrane	K_{Fb}
	A is catalyzing the membrane growth	α

In eq. 13 (see the main text - and in table **S1** here) k_f and η_f are the kinetic coefficients (the two reactions being supposed irreversible) of the chemical processes producing respectively A and B chemical species, K_{Fa} and K_{Fb} are the kinetic parameters of the passive transport (supposed ruled by the Fick's law), and α is the kinetic coefficient coupling the chemical species A with the container.

Note that the K_{F^*} coefficient includes the thickness of the membrane and a form factor due to the shape of the cell. Indeed, starting from the Fick's law:

$$\frac{dA}{dt} = \frac{D_A K_A}{\delta} S([A^*] - [A]) \qquad \text{S1}$$

where D_a and K_a are respectively the coefficient of diffusion and the partition coefficient, and S is the surface through the flow happens (the surface of the protocell), we can pass to concentrations by dividing with the actual volume of the protocell:

$$\frac{d[A]}{dt} = \frac{D_A K_A}{\delta V} S([A^*] - [A]) \qquad \text{S2}$$

and, because of we are focusing on spherical cells, we can obtain

$$\frac{d[A]}{dt} = \frac{D_A K_A}{\delta V^{\frac{1}{3}}} (36\pi)^{\frac{1}{3}}([A^*] - [A]) = \frac{K_{F_A}}{\delta V^{\frac{1}{3}}} ([A^*] - [A]) \qquad \text{S3}$$

Eq. 14:

Table S2 Description of the physic/chemical situation supposed in eq.14 (see the main text)

Physic/chemical situations	Description	Parameters
**Internal reactions**	A+B→A:B	k_f
	A:B + $\overline{F_A}$→2A+B	k_2
	A+B→A:B'	η_f
	A:B' + $\overline{F_B}$→A+2B	k_2'
**Interactions with the container**	The transmembrane flows of F_A and F_B are flexible and without bunds, in order to maintain the concentrations of F_A and F_B constant (so the symbols $\overline{F_A}$, $\overline{F_B}$)	$\overline{F_A}$ $\overline{F_B}$
	A is catalyzing the membrane growth	α

In eq. 14 (see the main text - and in table **S2** here) k_f and η_f are the kinetic coefficients of the chemical reactions producing the complexes that respectively contribute to the production of A and B chemical species (through the kinetic parameters k_2 and k_2' - all reactions being supposed irreversible); $\overline{F_A}$ and $\overline{F_B}$ are the constant concentration values of the chemicals F_A and F_B; α is the kinetic coefficient coupling the chemical species A with the container.

This framework is very effective in case of relatively high chemical concentrations; on the other hand it present difficulties when the concentrations are very low and the granularity of the system becomes significant.

So, the **second framework** directly simulates the disappearance and the formation of each single molecule by means of a Gillespie algorithm [35, 36], and replicates the same reactions. The Gillespie reaction constants can be derived from the more usual kinetic constant by means of the relations:

$$\begin{cases} Ccl_r = \frac{Kcl_r}{V} \\ Ccond1_d = \frac{Kcond1_d}{V} \\ Ccond1_d^{-1} = Kcond1_d^{-1} \\ Ccond2_d = \frac{Kcond2_d}{V} \end{cases} \qquad \text{S4}$$

K_{cond1}, K^{-1}_{cond1}, K_{cond2} are the kinetic constants of the complex formation, complex dissociation and complex final condensation (we are referring therefore to the nomenclature used in section 3).

In this case the integration with the container module (modelled by means of differential equation) is less obvious; nevertheless, the integration of these equations by means of the Euler schema can allow a very simple integration. Being a first order method this schema implies variable changes directly linked to a finite time interval: in our framework this time interval is directly derived from the Gillespie framework, which therefore becomes the main systems' engine. So the general schema is:

- While (simulation is non ended)
  - The Gillespie framework holds, and computes a finite time interval Δt_g
  - The simulation time is adjourned $t_s=t_s+\Delta t_g$
  - For each chemical x_i
    - the time passed from the chemical last change is adjourned $LC_i= LC_i +\Delta t_g$
    - its flows through the container is adjourned $fw_i= fw_i$ +f($rules$, LC_i)
    - if $|\text{int}(fw_i)-fw_i| \geq 1$, the number of molecules of x_i is varied of int(fw_i), fw_i is decremented by int(fw_i) and LC_i is set to 0

Int(X) and f($rules$, LC_i) indicate respectively the not fractional part of X and the dynamical rules (derived from of the differential equations description) that drive the chemical i mass exchange with the external environment. The time interval Δt_g is very tiny (it correspond to the formation/disappearance of few molecules) and therefore is compatible with the Euler schema.

By the way, note that if the volume is time dependent (V=V(t)) and if the process is quasi-static (the volume variation is not too fast) the relations in equation S4 can allow the Gillespie simulation of systems where the container volume can vary in time (it is enough to take into consideration the volume changes and recalculate the Gillespie reaction stochastic constants during the simulation). See also [58] for further details.

A note on simulations

These frameworks allow us great freedom of simulation. For simplicity reasons, however, in this paper we suppose that all condensations are symmetrical with respect to the two substrates. For example, in the case of the previous condensation, summarized by the reaction schema $x_a+x_b+x_c \rightarrow x_p+x_c$, we do not want to impose a particular order between the substrates: i.e., we suppose that one or the other one can equally be tied to the catalyst first.

To achieve this condition we simply impose that any condensation can occur in two symmetrical ways. By using the three steps presented on the paper, we have:

$x_a+x_c \rightarrow C_i$;	$x_b+x_c \rightarrow C_i$;
$C_i \rightarrow x_a+x_c$;	$C_i \rightarrow x_b+x_c$;
$x_b+C_i \rightarrow x_p+x_c$	$x_a+C_i \rightarrow x_p+x_c$

During the simulations corresponding to the cases discussed in the paper therefore each condensation is actually realized through two groups of different reactions, corresponding to the schemes proposed above. The values of the parameters presented on the paper correspond therefore to the kinetic coefficients of these two schemes, for symmetry supposed always equal.

Protocell split

For the sake of definiteness, in the simulations a value of $\xi=0.707$ has been chosen, which corresponds to the case of a spherical vesicle which breaks into two identical spherical vesicles. In all the three cases the width of the membrane is the same. The total quantity of lipids (i.e. the total volume of the lipid container) is the same, before and after the breakup. Details of the derivation can be found in [43]

Supplementary material S2

The stochastic simulator

The simulations in the paper are produced using two software tools: "proto_gill_tau" which simulates the evolution in time of a protocell, and “proto_gill_generazioni" which launches "proto_gill_tau" several times, gradually modifying the initial "proto_gill_tau" files that each new launch corresponds to the simulation of one of the two child cells of the previous launch.

proto_gill_tau

"proto_gill_tau" is able to simulate chemical reactions in two different physical environments: the CSR and the protocell. The software uses a stochastic dynamic, according to the Gillespie algorithm. The particular version “Tau leaping” was implemented to speed up the simulations [37]. "proto_gill_tau" needs four input files, and produces 8 output files. Below we report a general guide, and the details of the main files.

Input files (proto_gill_tau)

We show below a figure in which the flow of information of the “proto_gill_tau" software is schematized, and a table which shows the name of the input files and a brief description of them. Eventually, we describe in detail the two main files.

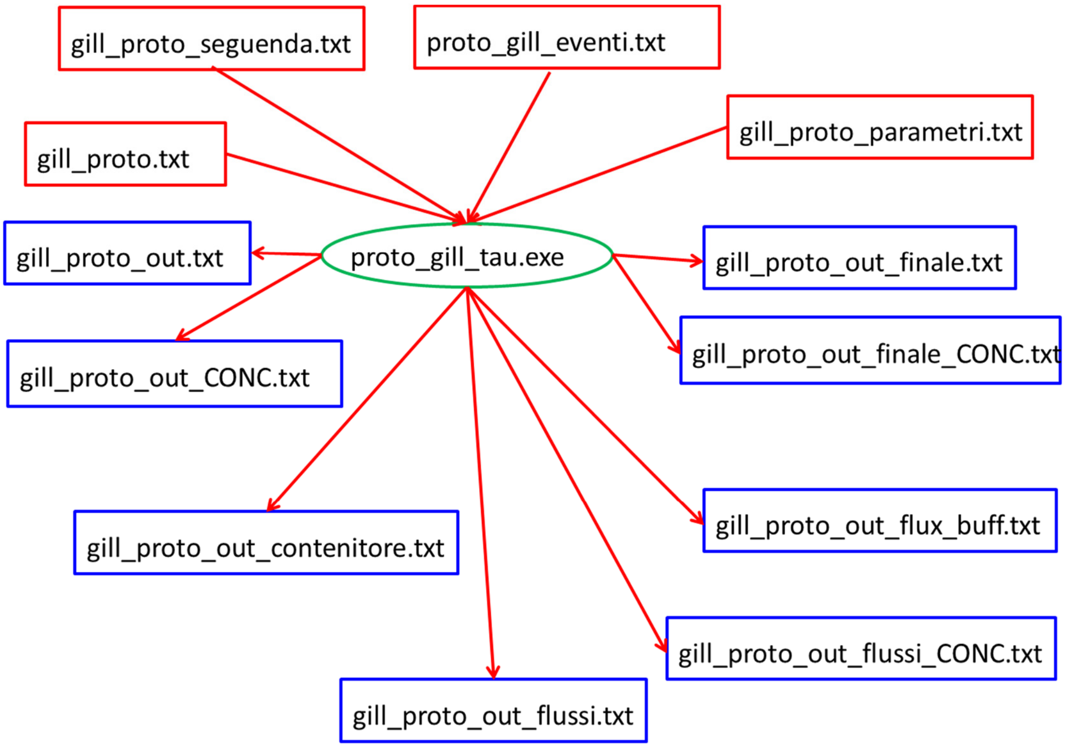

Figure S3 Flow of information of the "proto_gill_tau" software: in red the input files and in blue the output files

Table S3 The name of the input files and a brief description of them

Input file	General file description
gill_proto_parametri.txt	General control parameters
gill_proto.txt	File containing the details of the physical environment (CSTR or protocell) and the chemistry to be simulated
proto_gill_eventi.txt	File containing the sequence of events that could take place during the time evolution of a protocell. Currently only type "1" events (change of the number of molecules of the indicated chemical species) are implemented. A number of events equal to 0 (even in presence of rows describing some events) is acceptable and indicates "no events"
gill_proto_seguenda.txt	File containing the number and the indexes of some particular chemical species (and of some particular flux) the user is interested in

Description of the “gill_proto_parametri.txt” file

Table S4 Sequence and description of the parameters contained in “gill_proto_parametri.txt” file, with some example values

Typical value	Description
60000	maximum duration of the simulated event (seconds)
15000000000	maximum number of algorithm steps
10000	number of steps between two successive screen displays
10	number of simulated second between two successive entries of data on the output files
2789	Random seed
1	choice of physical environment<br>1: protocell; 0: CSTR
2.0	division threshold (the final lipid quantity, divided by the initial lipid quantity) - parameter active only in case of simulation of protocell

Description of the “gill_proto.txt” file

The "gill_proto.txt" file collects all the details of the chemistry to be simulated, and is therefore the most complicated file. Its basic structure is however not excessively complex.

The first three rows of the file "gill_proto.txt" indicate:

(i) the number of possible chemical reactions inside the container - protocell or CSTR
(ii) the number of membrane events - in the case some chemical species are able to cross the container wall
(iii) the number of chemical species involved

The list of the names of all chemical species present in the chemistry follows. To each name is associated:

1. the number of molecules present
2. a code that indicates the dynamical situation of the chemical species
   a. “0” indicates the default (a situation in which the chemical species can vary the number of its molecules, based on the events that occur in the system
   b. “1” codes for "keep constant the number of molecules" (in case of internal chemical species)
   c. “-2” codes for "keep constant the number of molecules" (in case of external chemical species)
3. the value of the coupling constant with the container - zero for all the species that do not catalyze the formation of the container
4. a string that reminds to the user the dynamical situation of the chemical species

Example:

- BBA 716 0 0.01 free_chemical_species
  - the chemical species "BA" has 716 molecules (a number that can change over time), and catalyzes the formation of the container with constant α=0.01

The software "proto_gill_tau" can simulate some types of chemical reactions. Each reaction has a code derived from the number of substrates and products:

- Code 10: 1 substrate, no products (the chemical species that plays the role of substrate is disappearing)
- Code 11: 1 substrate, 1 product (the chemical species that plays the role of substrate has changed in the chemical species that plays the role of product)
- Code 12: 1 substrate, 2 products (the chemical species that plays the role of substrate gives rise to two products)
- Code 21: 2 substrates, 1 product (the chemical species that plays the role of substrate "fuse" in 1 product)
- Code 22: 2 substrates, 2 products (the chemical species that plays the role of substrate have changed in the chemical species that plays the role of products)
- Code 23: 2 substrates, 3 products (the chemical species that plays the role of substrate have changed in the chemical species that plays the role of products)
- Code 32: 3 substrates, 2 products (the chemical species that plays the role of substrate have changed in the chemical species that plays the role of products)
- Code 33: 3 substrates, 3 products (the chemical species that plays the role of substrate have changed in the chemical species that plays the role of products)

The "gill_proto.txt" file then shows the list of reactions that can occur in the system. In each row corresponding to a chemical reaction there are therefore

1. the code of the reaction
2. the names of X and Y product (where X and Y correspond to the first digit and the second digit of the reaction code, respectively)
3. a real number (the Gillespie kinetic constant related to the reaction).

We report below some reaction examples

- 21 BA BBB *BABBB 0.000396
- 12 *BABBB BA BBB 14.86
- 22 *BABBB AA AABA BBB 0.00396

The three reactions just shown represent an example of a condensation (BA+AA→AABA, catalyzed by BBB), subdivided into the three steps described in the paper. Note that at the end of the sequence the BBB number remains unchanged: BBB plays therefore the role of the catalyst of the "global" reaction (of which *BABBB is the complex).

To these reactions are added the membrane passing events (if any), regulated by the chemical potential (in the simple form of concentration gradient, described by Fick's law):

- Code 210: three chemical species are indicated, followed by a real number
  - Example
    - 210 A_EXT A A 8e-017
      - "A_EXT" is the name of the external chemical species
      - the first "A" is the name of the internal chemical species (the gradient between "A_EXT" and "A" determines the flow through the membrane)
      - The second "A" is the chemical species that receives the flow
      - The final real number is the kinetic parameter associated to the reaction. This parameter includes the thickness of the membrane and a form factor due to the shape of the cell. In this work we focus on spherical cells, so holds that $K = \frac{D_A K_A}{\delta}(36\pi)^{\frac{1}{3}}$ where D_a represents the coefficient of diffusion and K_a the partition coefficient

Through this type of reaction it is possible to simulate a large environment around the protocell, by maintaining invariant the concentration of the first chemical species.

Eventually, the file presents three rows:

1. the initial radius of the spherical container, in meters
2. the thickness of the membrane, in meters
3. the density of the constituents of the membrane (grams / liter)
4. the value of water flow through the container (active only for CSTR)

Output files (proto_gill_tau)

The internal structure of the files is very simple, and it is not necessary to describe it in detail. The only things worth mentioning are:

- for convenience there are files in which the units of measurement are concentrations (molarity), and files in which are reported directly the numbers of molecules
- We can name "flows" the number of molecules that each chemical reaction has subtracted from the reactants (or added to the products) in the time interval identified by the "gill_proto_parametri.txt" file, divided by the time interval in seconds
  - The same concepts holds for the membrane events (the flows that are passing through the membrane)
- We can group reactions in some "general" categories. The sum of the flows of all the reactions belonging to each category are recorded within the file "gill_proto_out_flux_buff.txt". The categories are:
  - R10: all reactions that have 1 , and no product (the chemical species degrades)
  - R12: all reactions that have only 1 and 2 products
  - R21: all reactions that have 2 s and only 1product
  - R22: all reactions that have 2 s and 2 products
  - R23: all reactions that have 2 s and 3 products
  - R32: all reactions that have 3 s and 2 products

Here below we indicate the file names, and give a description of their information content.

Table S5 The name of the output files of "proto_gill_tau" and a brief description of them

Output file	General file description
gill_proto_out.txt	File containing the time evolution of all chemical species (for each species, the number of molecules present)
gill_proto_out_CONC.txt	File containing the time evolution of all chemical species (the concentration – molarity - of each species)
gill_proto_out_contenitore.txt	File containing the time evolution of the container (volume and number of moles – no changes in case of CSTR).
gill_proto_out_finale.txt	File containing the duplication time and the final number of molecules of all chemical species
gill_proto_out_finale_CONC.txt	File containing the duplication time and the final concentration (molarity) of all chemical species
gill_proto_out_flussi.txt	File containing the time evolution of all fluxes present within the system (first all the flows through the membrane, then all the fluxes related to each reaction present on the system). Units: number of molecules for each time interval (the time interval is determined by the "gill_proto_parametri.txt" file)
gill_proto_out_flussi_CONC.txt	File containing the time evolution of all fluxes present within the system (first all the flows through the membrane, then all the fluxes related to each reaction present on the system). Units: mols for each time interval (the time interval is determined by the "gill_proto_parametri.txt" file)
gill_proto_out_flux_buff.txt	File containing the time evolution of some kind of fluxes present within the system (flux categories: R10, R12, R21, R22, R23, R32). Units: number of molecules for each time interval (the time interval is determined by the "gill_proto_parametri.txt" file)

proto_gill_generazioni

The software "proto_gill_generazioni" launches "proto_gill_tau" several times, gradually modifying the input files of "proto_gill_tau" so that each new launch corresponds to the simulation of one of the two child cells of the previous launch. It saves the files generated in each interaction in a special folder, definable by the user. In the folder it also creates some files that summarize the data generated during the different generations (see figure **S4**).

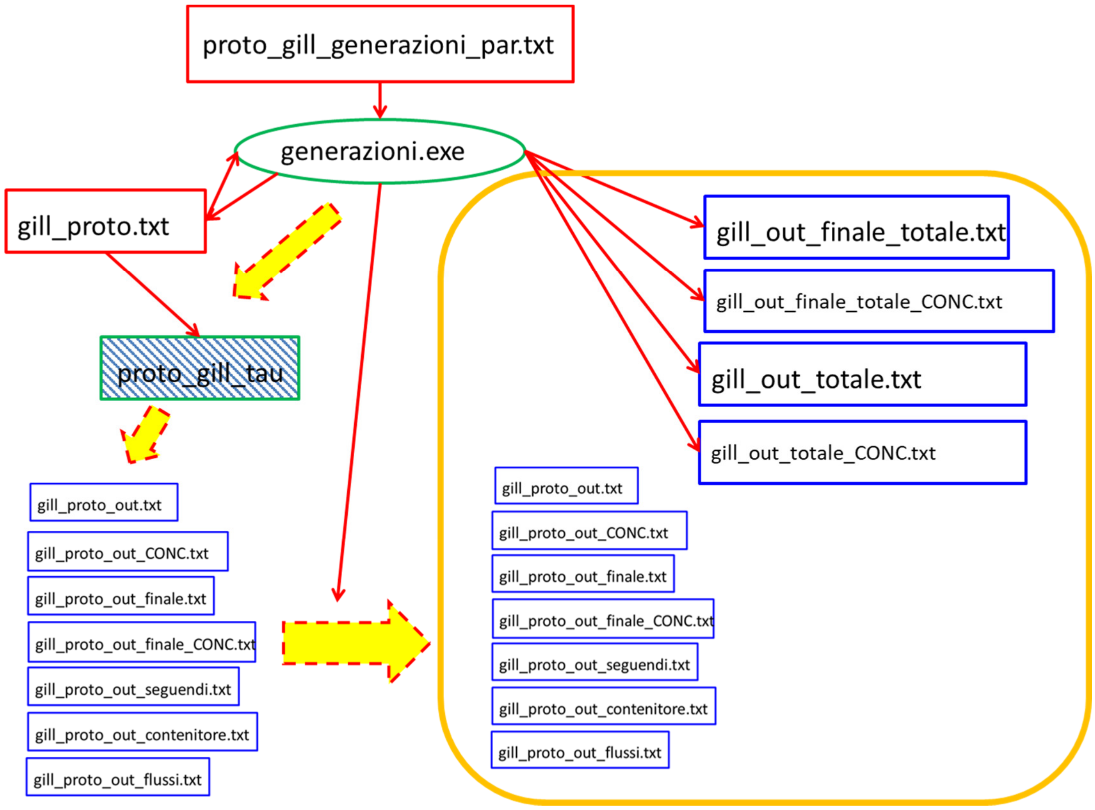

Figure S4 Flow of information of the “proto_gill_generazioni" software: in red the input files and in blue the output files. The tool launches the software "proto_gill_tau" several times, and saves the files generated in each interaction in a special folder, definable by the user. The four “biggest” blue files summarize the data generated during the different generations

Here below we indicate the input and the output file names that summarize the data generated during the different generations, and give a brief description of their content.

Input files (proto_gill_generazioni)

Description of the “proto_gill_generazioni_par.txt” file

Table S6 Sequence and description of the parameters contained in “proto_gill_generazioni_par.txt” file, with some example values

Typical value	Description
foldername	Name of the folder in which the user wish to save the data of the generations of protocells
20	Number of protocell generations
proto_gill_tau.exe	Name of the executable (implementing the simulation of protocells) to be launched

Output files (proto_gill_generazioni)

Table S7 The name of the output files of “proto_gill_generazioni” and a brief description of them

Output file	General file description
gill_out_finale_totale.txt	File containing the time evolution of all chemical species at duplication time (for each species, the number of molecules present)
gill_out_finale_totale_CONC.txt	File containing the time evolution of all chemical species at duplication time (the concentration – molarity - of each species)
gill_out_totale.txt	File containing the time evolution of all protocell generations: the first column therefore contains the value of the temporal step of each protocell, while the second column shows the absolute time, with the origin at the time of the departure of the first protocell. The time evolution of all chemical species is reported (for each species, the number of molecules present)
gill_out_totale_CONC.txt	File containing the time evolution of all protocell generations: the first column therefore contains the value of the temporal step of each protocell, while the second column shows the absolute time, with the origin at the time of the departure of the first protocell. The time evolution of all chemical species is reported (the concentration – molarity - of each species)

Supplementary material C

The deterministic simulator

The simulations in the paper are produced using two software tools: "proto_v1" which simulates the evolution in time of a protocell, and “proto_generazioni" which launches "proto_gill_tau" several times, gradually modifying the initial "proto_v1" files that each new launch corresponds to the simulation of one of the two child cells of the previous launch.

proto_v1

"proto_v1” is able to simulate chemical reactions in two different physical environments: the CSR and the protocell. The software uses a deterministic dynamic – in particular, an Euler algorithm with variable step. "proto_v1" needs three input files, and produces five output files. Below we report a general guide, and the details of the main files.

Input files (proto_v1)

We show below a figure in which the flow of information of the “proto_v1" software is schematized, and a table which shows the name of the input files and a brief description of them. Eventually, we describe in detail the two main files.

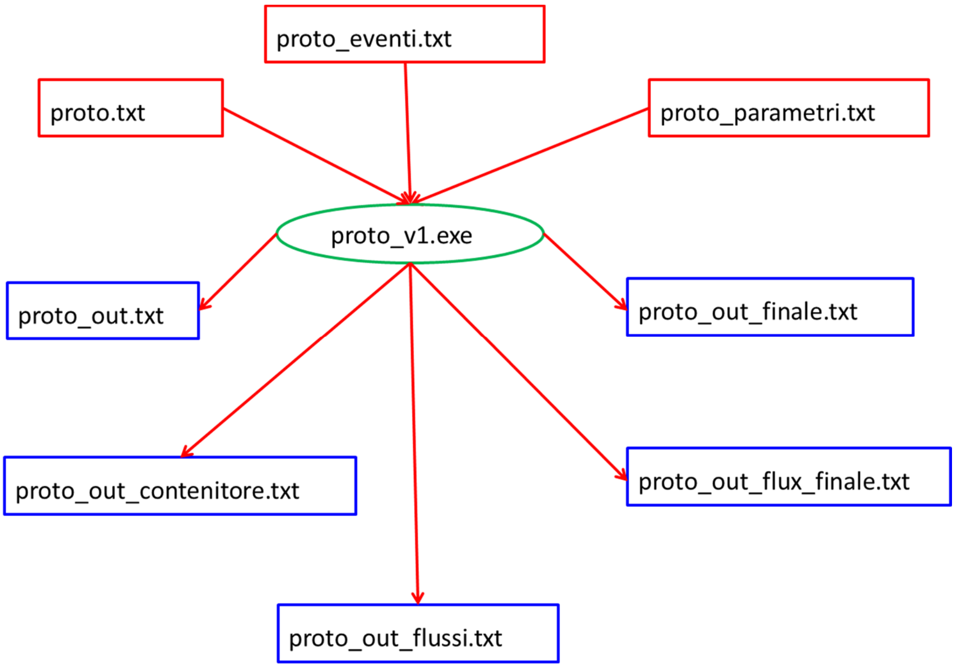

Figure S5 Flow of information of the “proto_v1" software: in red the input files and in blue the output files

Table S8 The name of the input files of the “proto_v1" software and a brief description of them

Input file	General file description
proto_parametri.txt	General control parameters
proto.txt	File containing the details of the physical environment (CSTR or protocell) and the chemistry to be simulated
Proto_eventi.txt	File containing the sequence of events that could take place during the time evolution of a protocell. Currently only type “1” events (change of the concentration of the indicated chemical species) are implemented. A number of events equal to 0 (even in presence of rows describing some events) is acceptable and indicates “no events”

Description of the “proto_parametri.txt” file

Table S9 Sequence and description of the parameters contained in “proto_parametri.txt” file, with some example values

Typical value	Description
60000	maximum duration of the simulated event (seconds)
2000000000	maximum number of algorithm steps
10000	number of steps between two successive screen displays
10	number of simulated second between two successive entries of data on the output files
1	choice of physical environment<br>1: protocell; 0: CSTR
2.0	division threshold (the final lipid quantity, divided by the initial lipid quantity) - parameter active only in case of simulation of protocell

Description of the “proto.txt” file

The "proto.txt" file collects all the details of the chemistry to be simulated, and is therefore the most complicated file. Its basic structure is however not excessively complex.

The first two rows of the file "gill_proto.txt" indicate:

(i) the number of possible chemical reactions
(ii) the number of chemical species involved

The list of the names of all chemical species present in the chemistry follows. To each name is associated:

1. the concentration (molarity) of molecules present
2. a code that indicates the dynamical situation of the chemical species
    a. “0” indicates the default (a situation in which the chemical species can vary the concentration, based on the events that occur in the system
    b. “1” codes for "keep constant the concentration"
3. the value of the coupling constant with the container - zero for all the species that do not catalyze the formation of the container
4. a string that reminds to the user the dynamical situation of the chemical species

Example:

- BBA 0.05 0 0.01 free_chemical_species
    - the chemical species "BA" has a concentration of 0.05M (a value that can change over time), and catalyzes the formation of the container with constant α=0.01

The software "proto_v1" can simulate some types of chemical reactions. Each reaction has a code derived from the number of substrates and products:

- Code 01: 0 substrate, 1 product (the injection of a substance from the outside is simulated)
- Code 10: 1 substrate, no products (the chemical species that plays the role of substrate is disappearing)
- Code 12: 1 substrate, 2 products (the chemical species that plays the role of substrate gives rise to two products)
- Code 21: 2 substrates, 1 product (the chemical species that plays the role of substrate “fuse” in 1 product)
- Code 22: 2 substrates, 2 products (the chemical species that plays the role of substrate have changed in the chemical species that plays the role of products)
- Code 23: 2 substrates, 3 products (the chemical species that plays the role of substrate have changed in the chemical species that plays the role of products)
- Code 32: 3 substrates, 2 products (the chemical species that plays the role of substrate have changed in the chemical species that plays the role of products)
- Code 33: 3 substrates, 3 products (the chemical species that plays the role of substrate have changed in the chemical species that plays the role of products)

The “proto.txt" file then shows the list of reactions that can occur in the system. In each row corresponding to a chemical reaction there are therefore

4. the code of the reaction
5. the names of X and Y product (where X and Y correspond to the first digit and the second digit of the reaction code, respectively)
6. a real number (the kinetic constant related to the reaction).

We report below some reaction examples

- 21 BA BBB *BABBB 1000
- 12 *BABBB BA BBB 14.86
- 22 *BABBB AA AABA BBB 500

The three reactions just shown represent an example of a condensation (BA+AA→AABA, catalyzed by BBB), subdivided into the three steps described in the paper. Note that at the end of the sequence the BBB number remains unchanged: BBB plays therefore the role of the catalyst of the “global” reaction (of which *BABBB is the complex).

To these reactions are added the membrane passing events (if any), regulated by the chemical potential (in the simple form of concentration gradient, described by Fick's law):

- Code 210: three chemical species are indicated, followed by a real number
  - Example
    - 210 A_EXT A A 8e-017
      - “A_EXT” is the name of the external chemical species
      - the first “A” is the name of the internal chemical species (the gradient between “A_EXT” and “A” determines the flow through the membrane)
      - The second “A” is the chemical species that receives the flow
      - The final real number is the kinetic parameter associated to the reaction. This parameter includes the thickness of the membrane and a form factor due to the shape of the cell. In this work we focus on spherical cells, so holds that $K = \frac{D_A K_A}{\delta}(36\pi)^{\frac{1}{3}}$ where D_a represents the coefficient of diffusion and K_a the partition coefficient

Through this type of reaction it is possible to simulate a large environment around the protocell, by maintaining invariant the concentration of the first chemical species.

Eventually, the file presents three rows:

1. the initial radius of the spherical container, in meters
2. the thickness of the membrane, in meters
3. the density of the constituents of the membrane (grams / liter)
4. the value of water flow through the container (active only for CSTR)

Output files (proto_v1)

The internal structure of the files is very simple, and it is not necessary to describe it in detail. The only things worth mentioning are:

- We can name "flows" the quantities (in mol) that each chemical reaction has subtracted from the reactants (or added to the products) in the time interval identified by the "gill_proto_parametri.txt" file, divided by the time interval in seconds
    - The same concepts holds for the membrane events (the flows that are passing through the membrane)

Here below we indicate the file names, and give a description of their information content.

Table S10 The name of the output files of "proto_gill_tau" and a brief description of them

Output file	General file description
proto_out.txt	File containing the time evolution of all chemical species (the concentration – molarity - of each species)
proto_out_contenitore.txt	File containing the time evolution of the container (volume and number of moles – no changes in case of CSTR).
proto_out_finale.txt	File containing the duplication time and the final concentration (molarity) of all chemical species
proto_out_flussi.txt	File containing the time evolution of all fluxes present within the system (first all the flows through the membrane, then all the fluxes related to each reaction present on the system). Units: mols for each time interval (the time interval is determined by the "proto_parametri.txt" file)
proto_out_flux_finale.txt	File containing the duplication time and the final values of all fluxes present within the system (first all the flows through the membrane, then all the fluxes related to each reaction present on the system). Units: mols for each time interval (the time interval is determined by the "proto_parametri.txt" file)

proto_generazioni

The software "proto_generazioni" launches "proto_v1" several times, gradually modifying the input files of "proto_v1" so that each new launch corresponds to the simulation of one of the two child cells of the previous launch. It saves the files generated in each interaction in a special folder, definable by the user. In the folder it also creates some files that summarize the data generated during the different generations (see figure **S6**).

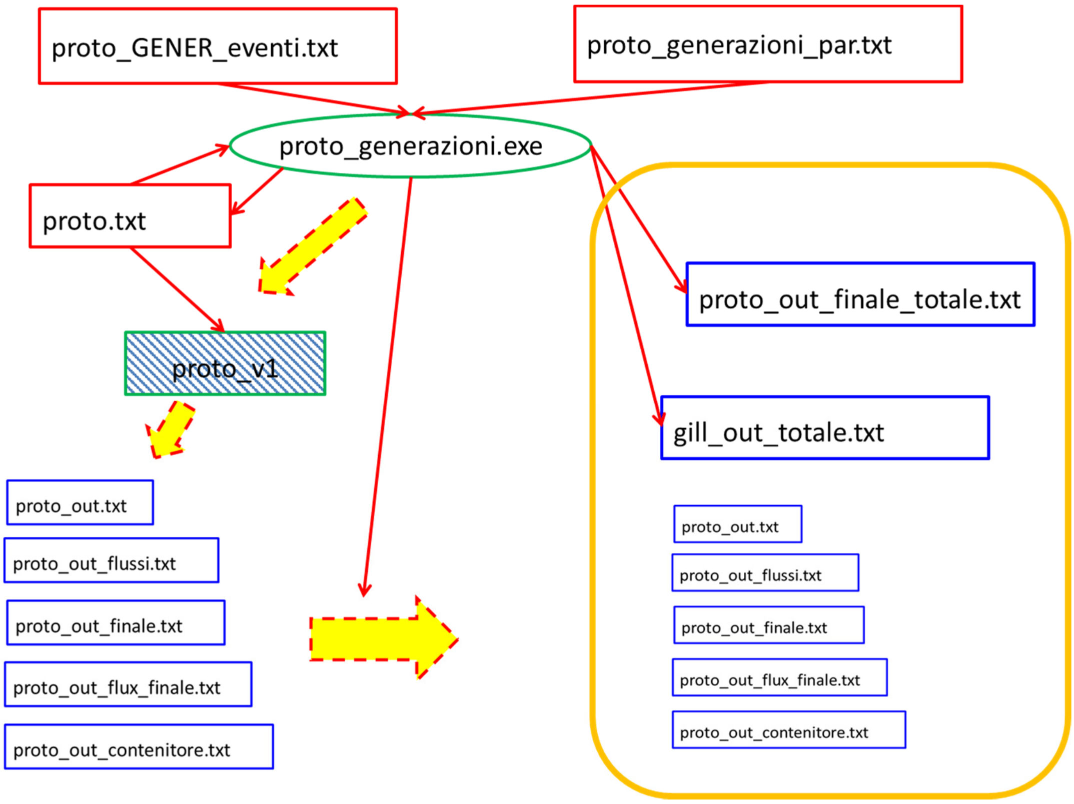

Figure S6 Flow of information of the “proto_generazioni" software: in red the input files and in blue the output files. The tool launches the software "proto_v1" several times, and saves the files generated in each interaction in a special folder, definable by the user. The two “biggest” blue files summarize the data generated during the different generations

Here below we indicate the input and the output file names that summarize the data generated during the different generations, and give a brief description of their content.

Input files (proto_generazioni)

Description of the “proto_generazioni_par.txt” file

Table S11 Sequence and description of the parameters contained in “proto_generazioni_par.txt” file, with some example values

Typical value	Description
foldername	Name of the folder in which the user wish to save the data of the generations of protocells
20	Number of protocell generations

Description of the “proto_GENER_eventi.txt” file

File containing the sequence of events that could take place during the division of a protocell. Currently only type “1” events (change of the concentration of the indicated chemical species) are implemented. A number of events equal to 0 (even in presence of rows describing some events) is acceptable and indicates “no events”

Output files (proto_generazioni)

Table S12 The name of the output files of “proto_generazioni” and a brief description of them

Output file	General file description
proto_out_finale_totale.txt	File containing the time evolution of all chemical species at duplication time (the concentration – molarity - of each species)
proto_out_totale.txt	File containing the time evolution of all protocell generations: the first column therefore contains the value of the temporal step of each protocell, while the second column shows the absolute time, with the origin at the time of the departure of the first protocell. The time evolution of all chemical species is reported (for each species, the number of molecules present)
proto_out_flux_finale_totale.txt	File containing the flux values at duplication time (first all the flows through the membrane, then all the fluxes related to each reaction present on the system). Units: mols for each time interval (the time interval is determined by the “proto_parametri.txt” file)

The code was developed in C language, using the ECLIPSE environment, Version: Photon Release (4.8.0). Please read the README.txt file for more details.

Code and support material are available at the link:

http://morespace.unimore.it/marcovillani/software/

MIT License

Copyright (c) 2019 Marco Villani

Permission is hereby granted, free of charge, to any person obtaining a copy of this software and associated documentation files (the "Software"), to deal in the Software without restriction, including without limitation the rights to use, copy, modify, merge, publish, distribute, sublicense, and/or sell copies of the Software, and to permit persons to whom the Software is furnished to do so, subject to the following conditions:

The above copyright notice and this permission notice shall be included in all copies or substantial portions of the Software.

THE SOFTWARE IS PROVIDED "AS IS", WITHOUT WARRANTY OF ANY KIND, EXPRESS OR IMPLIED, INCLUDING BUT NOT LIMITED TO THE WARRANTIES OF MERCHANTABILITY, FITNESS FOR A PARTICULAR PURPOSE AND NONINFRINGEMENT. IN NO EVENT SHALL THE AUTHORS OR COPYRIGHT HOLDERS BE LIABLE FOR ANY CLAIM, DAMAGES OR OTHER LIABILITY, WHETHER IN AN ACTION OF CONTRACT, TORT OR OTHERWISE, ARISING FROM,OUT OF OR IN CONNECTION WITH THE SOFTWARE OR THE USE OR OTHER DEALINGS IN THE SOFTWARE.
